# Comparative analysis of grape berry microbiota uncovers sour rot associates from a Maryland vineyard

**DOI:** 10.1371/journal.pone.0314397

**Published:** 2025-02-06

**Authors:** Blaise Jumbam, Magaly Toro, Mengjun Hu

**Affiliations:** 1 Department of Plant Science and Landscape Architecture, University of Maryland, College Park, Maryland, United States of America; 2 Joint Institute for Food Safety and Applied Nutrition (JIFSAN), University of Maryland, College Park, Maryland, United States of America; National Taiwan University, TAIWAN

## Abstract

Grape sour rot (GSR) is a disease complex involving fungi and bacteria that can cause significant yield losses of susceptible varieties. It is widely spread in the eastern U.S. and other grape-growing regions globally. Previous studies suggest that damaged fruit skin and feeding insects like *Drosophila* spp. are required for the disease to occur. Current control strategies for the management of sour rot are not sustainable, and research on the implications of chemical management of the disease on microbiome diversity is scarce. Our aim was to: i) investigate the effect of insecticide application and netting treatment on the microbiota of GSR-susceptible and tolerant grape varieties; and ii) identify the core microbial assemblages potentially associated with grape sour rot development in Maryland. Using a combined analysis of culture-dependent and independent data, we found that microbiota diversity of healthy grape berries did not change with netting, insecticide application, and between varieties. There was a significant difference in bacterial diversity between healthy and sour rot-affected berries. *Komagataeibacter* was consistently associated with infected berries followed by *Acetobacter* and *Gluconobacter*. This is the first study to report the association of *Komagataeibacter* with GSR-infected berries. It is thus imperative to investigate its role alongside that of other identified core microbiomes in sour rot development. *Candida* and *Pichia* were also consistent genera in infected berries. Several unidentified *Candida*, *Pichia*, and other fungal species from infected berries formed the core mycobiomes and it would be worth investigating their involvement in GSR development in Mid-Atlantic vineyards.

## Introduction

Plants recruit a variety of microorganisms that live in association with each of their organs in the latent or active states, either as endophytes or epiphytes [1]. These microbes play a multitude of roles, ranging from transforming soil organic matter into easily absorbable forms to improving the organoleptic properties of plant products such as wine and preventing the growth and activity of plant pathogenic fungi [2–4]. For instance, the combined use of *Saccharomyces cerevisiae* strains and non-*Saccharomyces* species like *Botrytis cinerea* can result in the production of highly improved Italian passito wines [5]. Interactions between microbes can also produce negative effects on their hosts. The development of grape sour rot (GSR) and the reduction of the quality of wine grapes are a result of interactions between certain microbes [6, 7]. GSR is a polymicrobial disease complex of grapevines that has been widely reported in grape-growing temperate regions. It is prevalent in wet, humid areas such as the Eastern United States [8]. The impacts of GSR are more detrimental to late-ripening, thin-skinned varieties with compact bunches after véraison [9, 10]. GSR involves the interaction of acetic acid bacteria, several yeast species, *Drosophila* spp., and the grapevine host plant [11, 12]. A few earlier studies have also documented the association of filamentous fungi with sour rot. For instance, GSR was initially thought to be the culmination of *Botrytis* infection [13, 14]. Similarly, *Aspergillus carbonarius* was reported as the causal agent of table grape (*Vitis vinifera*) sour rot in California [15]. *Alternaria tenuissima*, *Fusarium proliferatum*, and *Aspergillus* spp. were recently isolated from GSR leachate in China [16].

Plant microbiomes can be influenced by various factors connected with the host, microbes, and the environment. Gao et al. found differences in the microbiome assembly of the upper stem epidermis of healthy and *Fusarium* wilt-affected chili pepper (*Capsicum annuum*) plants [17]. In the same study, fungal communities were more sensitive to the disease than their bacterial counterparts. In a related study, bacterial communities were influenced by variety when different potato varieties were exposed to drought conditions [18]. Hall et al. identified and characterized epiphytic microbiomes associated with sour rotting grapes from Finger Lakes and Modesto in the US and Tasmania in Australia [19]. They found minimal changes in the microbiomes between healthy and sour rot-affected berries of five interspecific *Vitis vinifera* hybrids [19]. Given the important role that microbiomes play in local wine flavors [20] and their reported differences between locations [19, 21], it is pivotal to investigate and characterize microbial communities associated with sour rot symptoms of regional grapevine varieties.

Current control strategies for the management of sour rot are not sustainable, and research on the implications of chemical management of the disease on microbiome diversity, though scarce, seems to be gaining attention. Field experiments suggest that the combined use of insecticides and antimicrobial applications can significantly reduce GSR [22–24]. However, these chemical treatments could possibly affect the diversity and function of the phyllosphere microbiota [25]. Recently, there have been efforts to understand the effect of chemicals on target and non-target phyllosphere microbiota [26, 27]. For example, Wang et al. found differences in bacterial assemblages between insecticide (CAP; Chlorantraniliprole)-treated and untreated bulk soil from an Oryza *sativa* paddy in China [28]. Anti-fungal and biocontrol agents did not significantly influence the richness and diversity of the grape phyllosphere microbiota in northern Italy [25]. In a comparative study in Austria, herbicide application was reported to negatively impact grapevine root mycorrhization while significantly increasing xylemic bacteria compared to mechanical weeding [29]. Further studies in Greece and China investigated the role of fungicides on the phyllosphere microbiota of wheat [30] and pepper [31, 32]. In the US, Kenney and Hall explored the effect of Mustang Maxx (zeta-cypermethrin) and OxiDate 2.0 (hydrogen dioxide) on the incidence and severity of sour rot in Missourian vineyards [33]. The effect of environmentally friendly fungicides on cucumber phyllopshere microbiomes has recently been investigated in Taiwan [34]. Additionally, Barata et al. showed that the protection of grapevine bunches from fruit flies (*Drosophila* spp.) prevented the spread of sour rot [35].

Based on current literature, there is a general knowledge gap on the effect(s) of insecticides and antimicrobials on grape phyllosphere microbiota in the Mid-Atlantic vineyards. Information on the comparative analysis of the diversity and ecology of microbiota in GSR-susceptible and tolerant *Vitis vinifera* varieties in the Mid-Atlantic region is lacking. It is therefore important to understand the diversity, community assemblage, and role of bacterial and fungal communities associated with GSR, which has been a rising issue in this region. This knowledge could have broader applications in other grape-growing areas. Identifying the primary causal agent(s) of GSR is essential to guiding the development of targeted antimicrobials and the design of appropriate management strategies. In this study, we aimed to *i) investigate the effect of insecticide application and netting treatment on the microbiota of GSR-susceptible and tolerant grape varieties; and ii) identify the core microbial assemblages potentially associated with grape sour rot development in Maryland*.

## Materials and methods

### Grape sample collection

The choice of *Vitis vinifera* varieties sampled for this study was guided by their observed susceptibilities to GSR development in the field. Cabernet Franc has been reported to be more tolerant to sour rot compared to Vidal Blanc and Merlot that are susceptible to GSR. Vidal Blanc and Merlot are less tolerant to sour rot due to their moderately loose clusters and medium skin thickness [36], which are liable to berry splitting. From here, we shall refer to Cabernet Franc as ‘tolerant variety’ and Vidal Blanc as ‘susceptible variety’. Samples were collected from a vineyard in Maryland with permission from the field owner. Permits were not required for the work since animals or human subjects were not involved. In August 2022, an experiment was set up in a vineyard in Queenstown (latitude 38.998°N, longitude -76.135°W), located 22 m below sea level, with the goal of comparing the core microbiota within and between grape varieties. This vineyard was commercially managed following non-organic principles. The vineyard was established in the 1980s on a surface area of 9 hectares large, and separated into blocks, each containing a specific variety. The varieties were grafted either onto a 3309 or 10114 rootstock. Among some of the varieties, Merlot was 20–28 years old, VB 10–12 years, and CF was 8 years of age.

To evaluate the effect of insecticide application and netting on microbiota diversity and composition, the tolerant and susceptible varieties were considered. The experiment was designed following a previously described method [35] with slight modifications. Grapevines were randomly selected and tagged according to treatments. Grapevines were either treated (+Max) or not treated (-Max) with insecticide (Mustang Maxx: zeta-cypermethrin; FMC Corporation). In another treatment, they were protected (+Net) or not protected (-Net) with a netting material (Elastic Top Strainers; The Home Depot). Weeds were managed with herbicides. The insecticide was applied with a backpack sprayer (Jacto PJB-20) at a rate of 468 liters per hectare every seven to ten days (for a total of 3 applications), beginning at 15 Brix until harvest. The experiment was replicated three times and treatments were arranged in a completely randomized design.

At harvest, three grape clusters were collected from each treatment, giving a total of 24 samples (12 per variety). Clusters were collected in sterile plastic zip-loc bags using sterile scissors (sterilized with 70% ethanol). Samples were transported to the laboratory in a refrigerated container and processed within 12 h. Because of the heterogeneous nature of grape clusters, 10 berries were randomly harvested from the anterior, posterior, and equatorial portions of each cluster, giving a total of 30 berries per treatment. The berries were then weighed, crushed, and the macerate was centrifuged at 1,000 g in a Sorvall ST 16 centrifuge (ThermoFisher, Osterode, Germany) for 10 min. The resulting grape juice was collected into 15 ml falcon tubes and stored at –20°C for further use.

To determine the core microbiota associated with sour rot development in Maryland, we randomly collected sour rot-affected bunches among our trials at harvest. For each grapevine that had sour rot, a healthy bunch and a sour rot-affected bunch were collected yielding a total of 34 samples (17 healthy and 17 infected bunches). Of these 17 samples, six were from Merlot, two from Cabernet Franc, and nine from Vidal Blanc. These were transported to the laboratory and processed as described above.

### Isolation and culturing of fungi and bacteria

Culturable bacteria and fungi were isolated by plating 100 μl of the grape juice on specialized artificial growth media. The media used included malt extract agar (MEA; 3% malt extract, 2% agar, 1000 ml distilled water), yeast extract, peptone, dextrose agar (YPDA; 1% yeast extract, 2% peptone, 2% dextrose, 2% agar, 1000 ml distilled water), yeast extract, peptone, mannitol agar (YPMA; 0.5% yeast extract, 0.3% peptone, 2.5% mannitol, 1.5% agar, 1000 ml distilled water), potato dextrose agar (PDA; 39 g potato dextrose agar, 1000 ml distilled water), or yeast malt extract agar (YMA; 3 g yeast extract, 3 g malt extract, 5 g peptone, 10 g glucose, 20 g agar, 1000 ml distilled water) in 10 cm petri plates. Plates were parafilmed and incubated in the dark at 24°C for 3–5 days. Plates were monitored daily for growth, and the resulting bacterial and fungal colonies were streaked onto YPMA and PDA plates, respectively. Pure cultures were stored at 4°C prior to molecular analysis. Isolates were cryopreserved for long-term storage at –80°C in 40% glycerol.

### Genomic DNA extraction and amplification

Genomic DNA extraction was performed as in [37, 38], with slight modifications. Briefly, after thawing the grape juice from –20°C, 5 ml were transferred into a new falcon tube and centrifuged at 6,960 g for 5 min in an Eppendorf 5430 high-speed centrifuge (Eppendorf, Enfield, CT, USA). The supernatant was discarded, and the pellet was resuspended in 200 μl of sterile distilled water (SDW). The mixture was then transferred into a 1.5 ml Eppendorf (Ep) tube containing sterile 2.4 mm metal bulk beads (VWR, Batavia, IL, USA) and shaken on a FastPrep–24 sample preparation system (M.P. Biomedicals, Irvine, CA, USA) at 5 rps twice for 45 s each time. The homogenized mixture was transferred into a 2 ml Ep and total gDNA was extracted using the Omega E.N.Z.A.^®^ HP Fungal DNA extraction kit (Omega Bio-tek, Inc., Pinnacle Way, Norcross, GA, USA) following the manufacturer’s instructions with slight modifications. To increase gDNA yield, the DNA columns were dried overnight after the second DNA wash, and 50 μl of elution buffer was added and centrifuged at top speed for 90 secs. The gDNA was stored at –20°C for downstream use.

The DNA purity and concentration were checked using 1% agarose gel electrophoresis and a NanoDrop One Spectrophotometer (Thermo Scientific, Madison, WI, USA). The internal transcribed spacer (ITS) primer pair—namely ITS1f (5′-CTTGGTCATTTAGAGGAAGTAA-3′) and ITS2r (5′-GCTGCGTTCTTCATCGATGC-3′)—was used to amplify the partial fungal ITS region to assess fungal communities [39, 40]. Bacterial communities were assessed by amplifying the V4 segment of the 16S rRNA gene region using the primers 515F (5′-GTGCCAGCMGCCGCGGTAA-3′) and 806R (5′-GGACTACHVGGGTWTCTAAT-3′) [41], following the earth microbiome project (EMP) PCR protocol (http://www.earthmicrobiome.org/emp-standard-protocols/16s/) with some modifications. The forward and reverse primers used for the first PCR reactions were modified as in the EMP protocol to contain the Illumina overhangs—5′-TCGTCGGCAGCGTCAGATGTGTATAAGAGACAG-3′ and 5′-GTCTCGTGGGCTCGGAGATGTGTATAAGAGACAG-3′ respectively. All PCR reactions were conducted using a 25 μl volume, including 12.5 μl of Taq RED Master Mix Kit (Genesee Scientific, El Cajon, CA, USA), 2.5 μl each of the forward and reverse primers, 2 μl of template DNA and 5.5 μl of molecular grade water. Thermal cycler conditions consisted of an initial denaturation at 98°C for 1 min, followed by 30 cycles of 98°C for 10 s, 50°C for 30 s, and 72°C for 30 s and a final extension at 72°C for 5 min.

### PCR product purification, library preparation, quantification, normalization, and sequencing

PCR products for 96 samples were purified using ExoSAP-IT™ PCR Product Cleanup Reagent (Thermo Fisher Scientific Inc, Waltham, MA, USA) following the manufacturer’s recommendations. Amplicon libraries were constructed with the cleaned PCR products by ligation using a Nextera XT DNA sample preparation kit with Illumina adapter indexes, sets A and B, following the manufacturer’s instructions (Illumina, California, USA). Indexes contained barcodes unique to each sample, allowing the identification of samples in a mixture for an Illumina sequencing run. The amplicons from each sample were quantified using the NanoDrop One Spectrophotometer and equimolar solutions were pooled together. Then, the library’s double DNA strand concentration was measured in a Qubit 3.0 fluorometer (Life Technologies, California, USA), and the molar concentration was adjusted to 1000 pM. The final library was sequenced with 40% PhiX on a NextSeq 1000 (Illumina) using the Illumina Nextseq P1 reagent cartridge with 600 cycles. Sequencing was performed at the food safety laboratory of JIFSAN at the University of Maryland, College Park. See supplementary materials for step-by-step summary from sampling to sequencing (S1 Appendix).

### Bioinformatic and sequence analysis

Output data from the NextSeq 1000 was analyzed with Quantitative Insights Into Microbial Ecology 2 (QIIME2 v2020.2) software [42]. Raw sequence reads were processed as in [37]. Briefly, sequences were checked for quality, and poor-quality reads (Q < 20) were trimmed. Chimeras were filtered out using the open-reference OTU picking algorithm DADA2 [43]. Filtered reads were then clustered into operational taxonomic units (OTUs) based on a 0.03% threshold (97% similarity) and classified using the Silva v.138.1 [44] and UNITE v.8.0 [45] reference databases for bacteria 16S rRNA and fungi ITS regions respectively. A phylogenetic tree was automatically constructed from a gap-filtered alignment using the QIIME2 plugin of FastTree [46]. Sequences that shared similarity of ≥97% were considered conspecific [47]. An OTU table was created, excluding unaligned sequences, singletons, and sequences matching plant plastids. Additionally, all OTUs belonging to the “Unclassified *Fungi*” and “Unclassified *Bacteria*” were further filtered by blasting against the NCBI search tool. All resulting OTUs that did not match fungi and bacteria were discarded. To reduce biases arising from different sequencing depths, sequence data were rarefied to the same depth for each sample before downstream analysis in R. Within sample diversity (alpha diversity) estimates were calculated using Faith’s phylogenetic diversity, Observed features (number of species), Shannon (species diversity), and Pielou’s evenness indexes. Between sample diversity (beta diversity) based on Bray Curtis distances were used to examine community dissimilarity and determine the impact of experimental factors on the bacterial and fungal community structure.

### Statistical analysis

Rarefied sequence data were examined for normality using the Shapiro-Wilk test for normality [48] (S1 Table). Kruskal-Wallis test of significance [49] was used to verify the relationships among sample data that were not normally distributed. Permutational multivariate analysis of variance (PERMANOVA) [50] with Bonferroni correction [51] was performed using ADONIS2 of the vegan package [52] in R to determine differences in microbial composition and how they clustered among varieties and experimental treatments. Non-metric Multidimensional Scaling (NMDS) [53] plots were used to visualize these differences. OTUs that significantly influenced the abundance levels in different treatments were determined using linear discriminant analysis (LDA) effect size (LEfSe) [54]. The core OTUs were defined as in Jumbam et al. [37] with slight modifications. Briefly, an OTU had to be present in 75% of samples under comparison after normalization of the data based on their z-score. The culturable and culture-independent data were pooled together to determine the core microbiotas. Abundance values were standardized by calculating their z-scores and heatmaps were generated to determine the core microbiotas associated with sour rot development. The R software v.4.3.2 [55] was used for downstream data analysis. Shared OTUs between treatments were visualized using Venn diagrams, and the core microbiota were examined using heatmaps.

### Data availability

The sequenced data used in this study have been deposited in the NCBI Sequence Read Archive (SRA) under the BioProject identifiers PRJNA1100499 and PRJNA1100588. The R code and input files are available at (https://github.com/BlaiseJumbam/winegrape_sour_rot_analysis/tree/main).

## Results

### Sequence read analysis

**Fig 1:**
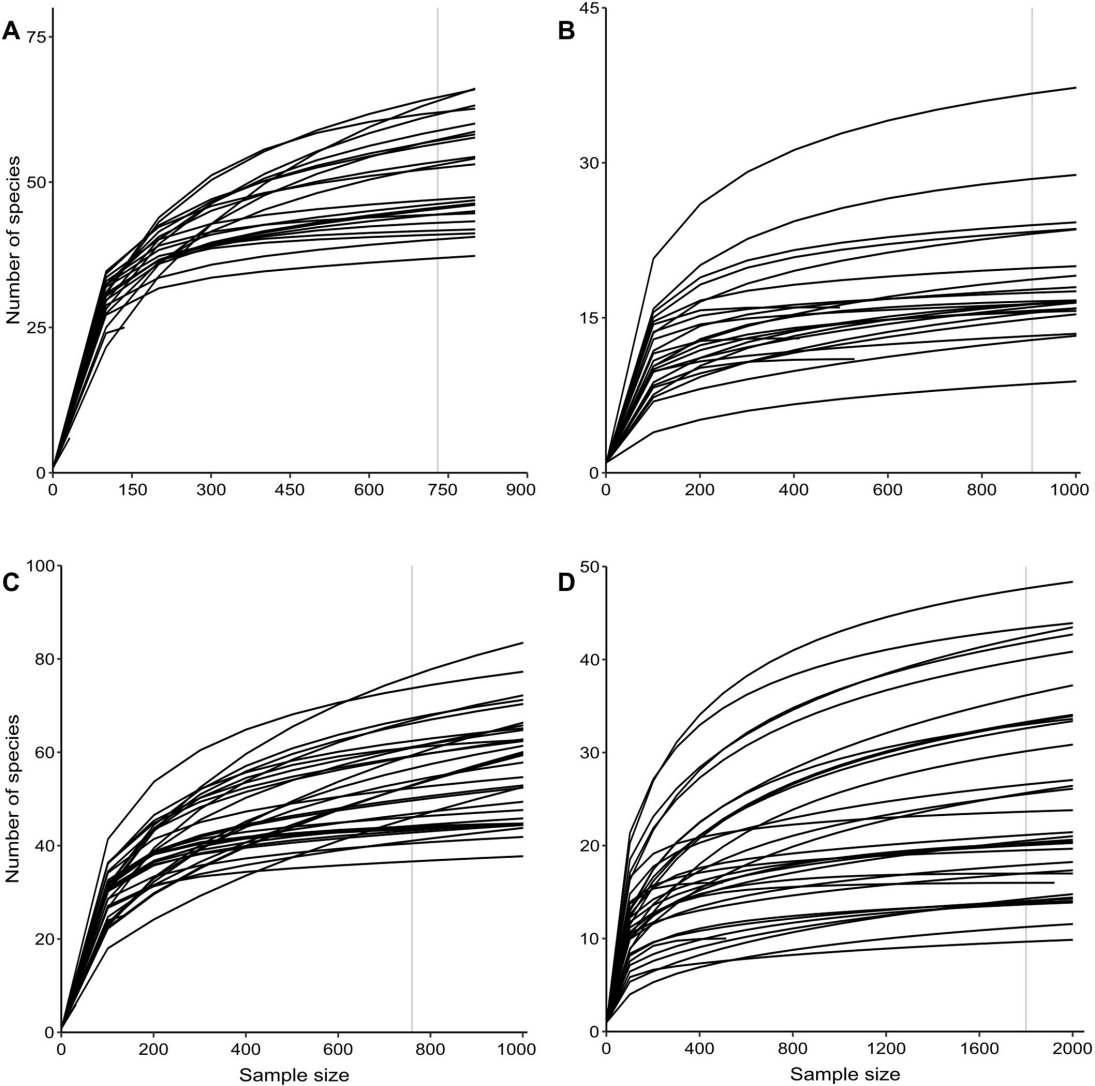
Rarefaction curves. *(A)* Bacterial samples from tolerant and susceptible varieties under insecticide and netting treatments. *(B)* Fungal samples from tolerant and susceptible varieties under insecticide and netting treatments. *(C)* Bacterial samples from tolerant and susceptible varieties under healthy and infected berry treatments. *(D)* Fungal samples from tolerant and susceptible varieties under healthy and infected berry treatments. The vertical gray line indicates the subsampling depth at which maximum diversity was captured and most samples were retained. The y-axis represents the number of species (OTUs) in each sample while the x-axis represents the number of sequences sampled.

For culture-independent data, the total number of reads for the 16S dataset used to investigate the effect of insecticide, netting, and variety on the microbiota of tolerant and susceptible grape berries was 3,156,316 with an average of 126,253 reads per sample. Meanwhile, the ITS dataset yielded a total of 19,947,694 reads with a mean of 797,908 reads per sample. After filtering the samples to drop all unclassified OTUs, the bacterial and fungal datasets were rarefied at 730 and 907 sequences respectively per sample to capture maximum diversity and minimize sample loss (Fig 1A and 1B). At these rarefaction thresholds, we lost three samples from the 16S dataset and two from the ITS dataset. There were 507 and 484 unique sequences for the 16S and ITS datasets, respectively. For the dataset on healthy and infected grape berries, there were a total of 6,886,200 reads for the 16S dataset, and each sample had an average of 196,749 reads. The ITS dataset, on the other hand, had 68,815,306 reads in total, with each sample having 1,966,152 on average. Sequence reads from all 16S datasets had an average length of 250 base pairs, while the average length of ITS sequences was 242 base pairs. After filtering to drop all unclassified OTUs, we subsampled the bacterial and fungal datasets at 760 and 1800 sequences per sample respectively (Fig 1C and 1D). At this subsampling depth, we lost three samples from the 16S dataset and two from the ITS dataset. These latter two datasets yielded 843 unique sequences for 16S and 1,213 for ITS. Finally, in the culture-based approach, we successfully isolated and Sanger-sequenced approximately 700 fungi and bacteria combined. Details of the total number of sequenced reads by sample for the culture-independent technique are given in S2 Table.

### Effect of insecticide application and netting on microbial communities of healthy tolerant and susceptible berries

#### Diversity of microbiomes associated with healthy berries from the tolerant and susceptible varieties

**Fig 2:**
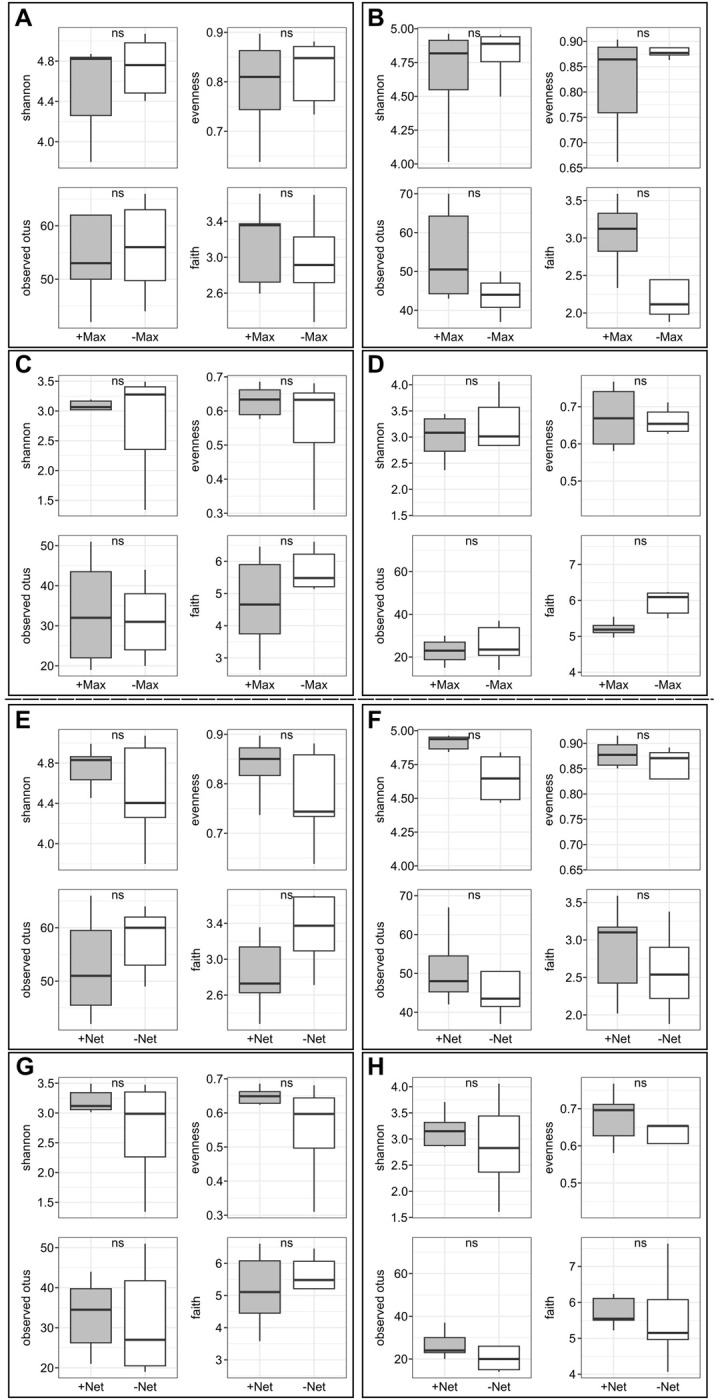
Alpha diversity measures of microbiota communities. *(A)* Bacterial communities under insecticide treatment for the tolerant variety. *(B)* Bacterial communities under insecticide treatment for the susceptible variety. *(C)* Fungal communities under insecticide treatment for the tolerant variety. *(D)* Fungal communities under insecticide treatment for the susceptible variety. *(E)* Bacterial communities under netting treatment for the tolerant variety. *(F)* Bacterial communities under netting treatment for the susceptible variety. *(G)* Fungal communities under netting treatment for the tolerant variety; and *(H)* Fungal communities under netting treatment for the susceptible variety.

The alpha diversity of bacterial and fungal communities on the two grape varieties was estimated using Shannon, Evenness, Observed OTUs, and Phylogenetic diversity indexes, as shown in Table 1. The average number of observed OTUs for bacterial communities was 53, and 30 were found for fungal communities. The results indicate that there was no difference in bacterial diversity between healthy insecticide-treated (+Max) and untreated (-Max) tolerant and susceptible grapes (Table 1; Fig 2A and 2B). Similar results were observed for fungal diversity between healthy +Max and -Max tolerant and susceptible grape berries (Table 1; Fig 2C and 2D). Additionally, we did not observe any differences in microbiota diversity between healthy net-protected (+Net) and unprotected (-Net) tolerant and susceptible grape berries for bacteria (Table 1; Fig 2E and 2F) and fungi (Table 1; Fig 2G and 2H). Considering the varietal factor, there were no significant differences in the bacterial or fungal species alpha diversity between the two varieties (S1 Fig).

**Table 1:** Analysis of variance among different treatments on two experimental varieties of *Vitis vinifera*.

Variety	Gene	Diversity Index				Treatment
		Evenness	Shannon	Faith	Observed OTUs	
Tole.	ITS	*KW =* .*231; p =* .*631*	*KW =* .*103; p =* .*749*	*F*_*1*,*10*_ *= 2*.*374; p =* .*154*	*F*_*1*,*10*_ *=* .*088; p =* .*773*	+Max/-Max
		*KW = 1*.*641; p =* .*200*	*KW =* .*410; p =* .*522*	*F*_*1*,*10*_ *=* .*008; p =* .*932*	*F*_*1*,*10*_ *= 0*.*061; p =* .*810*	+Net/-Net
	16S	*F*_*1*,*9*_ *=* .*343; p =* .*573*	*F*_*1*,*9*_ *=* .*901; p =* .*367*	*F*_*1*,*9*_ *=* .*404; p =* .*541*	*F*_*1*,*9*_ *=* .*150; p =* .*708*	+Max/-Max
		*F*_*1*,*9*_ *= 1*.*872; p =* .*204*	*F*_*1*,*9*_ *= 1*.*296; p =* .*284*	*F*_*1*,*9*_ *= 3*.*758; p =* .*084*	*F*_*1*,*9*_ *=* .*960; p =* .*353*	+Net/-Net
Susc.	ITS	*F*_*1*,*8*_ *=* .*460; p =* .*517*	*F*_*1*,*8*_ *=* .*009; p =* .*929*	*F*_*1*,*8*_ *= 1*.*435; p =* .*265*	*KW =* .*103; p =* .*748*	+Max/-Max
		*F*_*1*,*8*_ *= 1*.*053; p =* .*335*	*F*_*1*,*8*_ *=* .*499; p =* .*500*	*F*_*1*,*8*_ *=* .*052; p =* .*825*	*KW =* .*702; p =* .*402*	+Net/-Net
	16S	*KW =* .*409; p =* .*522*	*KW =* .*409; p =* .*522*	*F*_*1*,*8*_ *= 5*.*239; p =* .*051*	*F*_*1*,*8*_ *= 2*.*530; p =* .*150*	+Max/-Max
		*KW =* .*045; p =* .*831*	*KW = 2*.*909; p =* .*088*	*F*_*1*,*8*_ *=* .*498; p =* .*500*	*F*_*1*,*8*_ *=* .*112; p =* .*746*	+Net/-Net

Tole. = Tolerant variety, Susc. = Susceptible variety, +Max = Mustang Max treated, -Max = Mustang Max untreated, +Net = Bagged with net, -Net = Not bagged with net

#### Abundance of bacterial and fungal communities of healthy tolerant and susceptible berries

**Fig 3:**
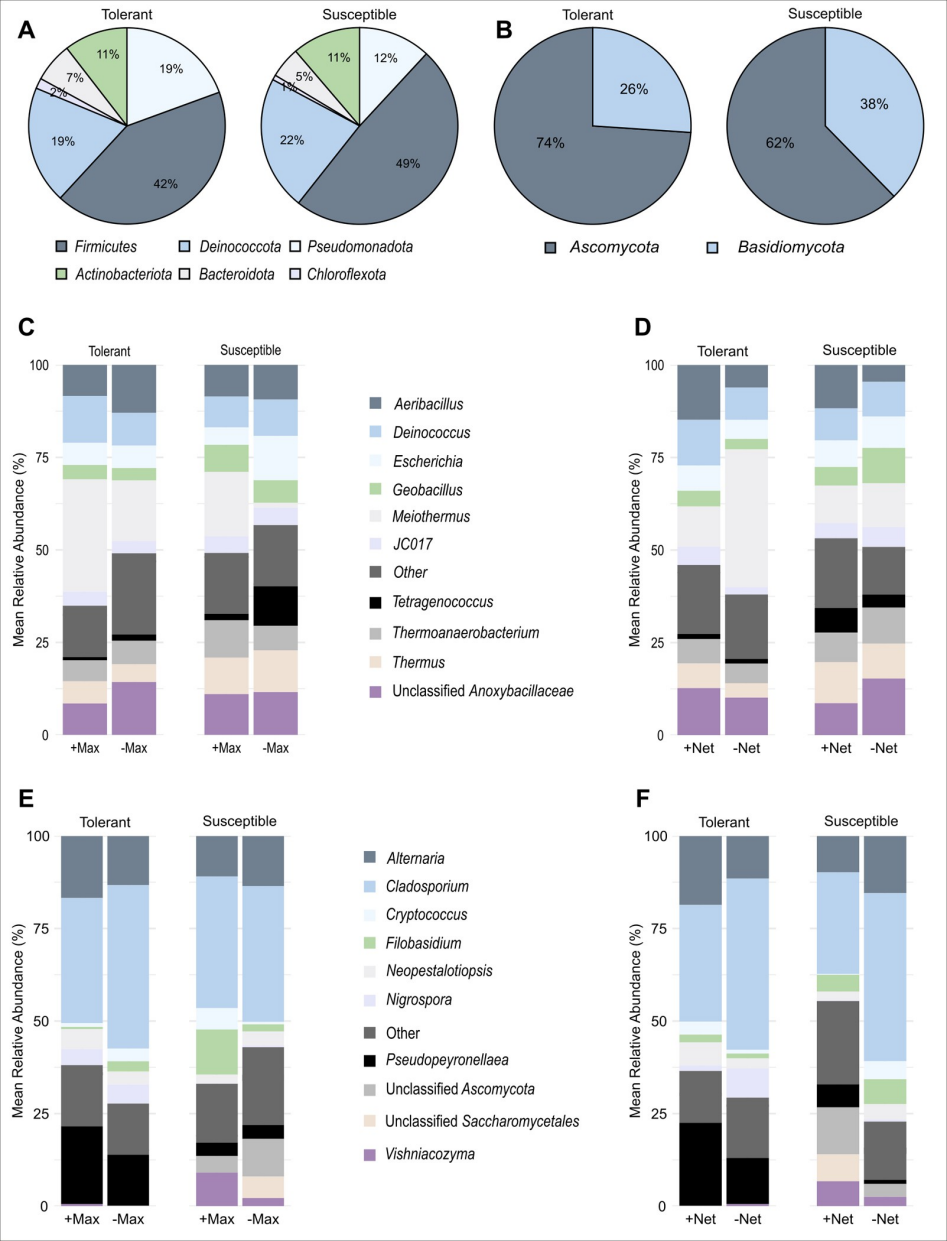
Relative abundances of recovered OTUs. *(A)* Bacterial phyla in the tolerant and susceptible varieties. *(B)* Fungal phyla in the tolerant and susceptible varieties. *(C)* Bacterial genera from insecticide treatments between the tolerant and susceptible varieties. *(D)* Bacterial genera from bagging treatments between the tolerant and susceptible varieties. *(E)* Fungal genera from insecticide treatments between the tolerant and susceptible varieties; and *(F)* Fungal genera from bagging treatments between the tolerant and susceptible varieties.

The alpha diversity analyses indicate that insecticide (+Max/-Max), netting (+Net/-Net), and varietal (S1 Fig) treatments did not have a significant effect on the microbial community diversity of berries from healthy tolerant and susceptible varieties at the end of the harvest season. However, there were differences in the community composition of various microbial phyla and genera (OTUs) recovered. Overall, we recovered 46 (7 unclassified) unique genera distributed in 35 (2 unclassified) families and 6 bacterial phyla. The bacterial community of the tolerant and susceptible varieties was dominated by *Firmicutes*, *Deinococcota*, *Pseudomonadota*, *Actinobacteriota*, and *Bacteroidota* (Fig 3A). These taxa accounted for 98 and 99% of total reads, respectively in the two varieties. However, the proportions of these phyla differed between the two varieties. There were more *Firmicutes* and *Deinococcota* in the susceptible (49%) than in the tolerant (41%) variety, while the reverse was true for *Pseudomonadota*. In total, we recovered 64 (14 unclassified) unique fungal genera distributed in 49 (8 unclassified) families within the two major fungal phyla (*Ascomycota* and *Basidiomycota*). The *Ascomycota* dominated *Basidiomycota* in both varieties (Fig 3B).

As observed for phyla, the abundance of bacterial and fungal genera also varied between varieties under insecticide and netting treatments. All bacterial and fungal genera that had a relative abundance below a predefined threshold to select for the top ten taxa were grouped into a category called “Other”. *Meiothermus* was more abundant in +Max berries than -Max berries for both varieties. There were more *Deinococcus* in +Max than in -Max berries and more *Aeribacillus* and “Unclassified *Anoxybacillaceae*” in -Max than +Max berries of the tolerant variety. For the susceptible variety, there were more *Thermoanaerobacterium* in +Max than -Max berries while *Escherichia* and *Tetragenococcus* were more abundant in -Max than +Max berries (Fig 3C). *Aeribacillus* was more abundant in +Net than -Net berries for both varieties. *Deinococcus*, *Escherichia*, *Geobacillus*, *Marinilabiliaceae bacterium (JC017)*, *Thermoanaerobacterium*, *Thermus*, and “Unclassified *Anoxybacillaceae*” were more abundant in +Net than -Net berries while *Meiothermus* was most abundant in -Net than +Net berries of the tolerant variety. On the other hand, *Tetragenococcus* and *Thermus* were most abundant in +Net than -Net berries and *Geobacillus*, *Thermoanaerobacterium* and “Unclassified *Anoxybacillaceae*” were more abundant in -Net than +Net berries of the susceptible variety (Fig 3D).

Among the top ten most abundant fungal genera, *Cladosporium* was the most abundant fungus across all treatments in the tolerant and susceptible varieties (Fig 3E and 3F). *Alternaria*, *Neopestalotiopsis*, *Pseudopeyronellaea*, and *Vishniacozyma* were more abundant in +Max berries while *Cladosporium*, *Cryptococcus* and *Filabasidium* were more abundant in -Max berries of the tolerant variety (Fig 3E). On the other hand, *Cryptococcus*, *Filabasidium*, and *Vishniacozyma* were more abundant in +Max berries while the abundance of *Alternaria*, *Neopestalotiopsis*, *“*Unclassified *Ascomycota”*, and *“*Unclassified *Ascomycota”* was higher in -Max berries of the susceptible variety. The abundance of *Cladosporium* was similar in both +Max and -Max berries of the latter variety (Fig 3E). A similar trend was observed for fungal genera in +Net and -Net berries for both varieties. *Alternaria*, *Cryptococcus*, *Filobasidium*, *Neopestalotiopsis*, and *Pseudopeyronellaea* were abundant in +Net berries while *Cladosporium*, *Nigrospora*, and *Vishniacozyma* were relatively more abundant in -Net berries of the tolerant variety (Fig 3F). Finally, *Pseudopeyronellaea*, *“*Unclassified *Ascomycota”*, *“*Unclassified *Saccharomycetales”*, and *Vishniacozyma* were abundantly present in +Net berries while *Alternaria*, *Cladosporium*, *Cryptococcus*, *Filobasidium*, and *Neopestalotiopsis* were more abundant in -Net berries of the susceptible variety (Fig 3F). Overall, the abundance of *Vishniacozyma* was very low in the tolerant compared to the susceptible variety.

#### Beta diversity and linear discriminant analysis effect size (LEfSe) of microbiota assemblages in tolerant and susceptible varieties

**Fig 4:**
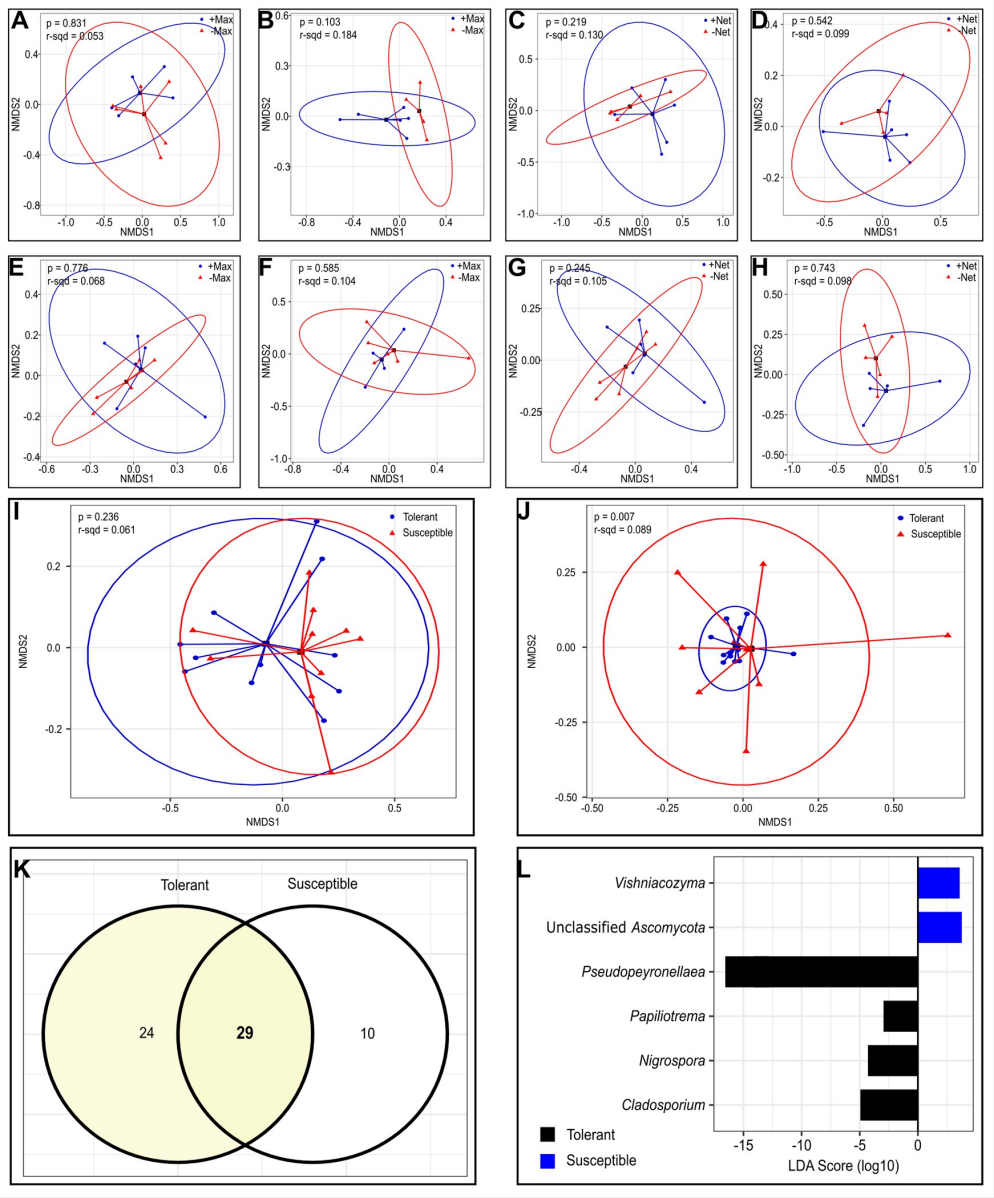
Healthy CF and VB samples showed varied differences in microbial community composition. *(A–B)* Bacterial community clusters between +Max/-Max for tolerant and susceptible varieties. *(C–D)* Bacterial community clusters between +Net/-Net for tolerant and susceptible varieties. *(E–F)* Fungal community clusters between +Max/-Max for tolerant and susceptible varieties. *(G–H)* Fungal community clusters between +Net/-Net for tolerant and susceptible varieties. *(I)* NMDS for bacteria between varieties based on Bray-Curtis distances. *(J)* NMDS for fungi between varieties based on Bray-Curtis distances. *(K)* Distinct fungal OTUs between varieties*; and (L)* LEfSe plots showing fungal OTUs responsible for the observed differences in community composition between varieties. The bars represent the effect size (LDA) for a particular taxon in a certain treatment group. The length of the bar represents a log10 transformed LDA score and the colors illustrate which group that taxon was found to be more abundant compared to the other group.

Non-metric multidimensional scaling (NMDS) analysis was used to assess how the microbiota clustered between insecticide and net treatments for each variety. Beta diversity analysis using Bray-Curtis distances showed that there was no distinction in bacterial community clusters between +Max/-Max berries from tolerant (PERMANOVA *F*_*1*,*9*_ *= 0*.*509*, *p = 0*.*831*) and susceptible (PERMANOVA *F*_*1*,*8*_ *= 1*.*806*, *p = 0*.*103*) varieties (Fig 4A and 4B respectively). Similarly, the NMDS analysis indicated that bacterial communities were not significantly different between +Net/-Net berries in the tolerant (PERMANOVA *F*_*1*,*9*_ *= 1*.*347*, *p = 0*.*219*) and susceptible (PERMANOVA *F*_*1*,*8*_ *= 0*.*877*, *p = 0*.*542*) varieties (Fig 4C and 4D respectively).

Like bacterial, fungal communities were also not significantly different between +Max/-Max berries for the tolerant (PERMANOVA *F*_*1*,*10*_ *= 0*.*727*, *p = 0*.*776*) and susceptible (PERMANOVA *F*_*1*,*8*_ *= 0*.*933*, *p = 0*.*585*) varieties (Fig 4E and 4F respectively). In a similar way, there were no significant differences in fungal communities between +Net/-Net berries for both the tolerant (PERMANOVA *F*_*1*,*10*_ *= 0*.*169*, *p = 0*.*245*) and susceptible (PERMANOVA *F*_*1*,*8*_ *= 0*.*867*, *p = 0*.*743*) varieties as OTUs clustered together (Fig 4G and 4H respectively). S3 Table shows complete PERMANOVA results of treatments separated by grape varieties. We also performed NMDS analysis for bacterial and fungal communities between the two varieties. The results indicated that bacterial communities showed no differences between varieties (Fig 4I, Table 2). On the contrary, there was a significant difference in fungal communities between the two varieties (Fig 4J, Table 2).

**Table 2:** Permutational multivariate analysis of variance for bacteria and fungi between tolerant and susceptible *Vitis vinifera* varieties.

Gene	Factor	Degrees of Freedom	Sum of Squares	R^2^	F	*p*
16S	Variety	1	0.215	0.061	1.228	0.236
	Residual	19	3.333	0.939		
	Total	20	3.548	1.000		
ITS	Variety	1	0.463	0.089	1.958	**0.007**
	Residual	20	4.725	0.911		
	Total	21	5.188	1.000		

There was a total of 63 distinct fungal OTUs recovered from the two varieties of which 29 were shared while 24 and 10 OTUs were unique to the tolerant and susceptible varieties respectively (Fig 4K). Linear discriminant analysis effect size was employed to determine the taxa (OTUs and other taxonomic levels) responsible for the observed differences in relative abundances of fungal microbiota between varieties. Two fungal OTUs belonging to *Vishniacozyma* (otu16) and *“*Unclassified *Ascomycota”* (otu23) (Fig 4L; Table 3) were significantly more abundant in berries from the susceptible variety. On the other hand, seven fungal OTUs belonging to *Cladosporium* (otu1), *Nigrospora* (otu7), *Papiliotrema* (otu70), and *Pseudopeyronellaea* (otus3, 4, 6, and 12) were significantly most abundant in berries from the tolerant variety (Fig 4L, Table 3).

**Table 3:** Linear discriminant analysis results showing significantly different fungal taxa among two grape varieties under different treatments.

Gene	OTU	Treatment	LDA	*p*-value	Phylum	Genus
ITS	otu1	Tolerant	4.946	0.025	*Ascomycota*	*Cladosporium*
	otu7	Tolerant	4.297	0.008	*Ascomycota*	*Nigrospora*
	otu70	Tolerant	2.953	0.012	*Basidiomycota*	*Papiliotrema*
	otu3	Tolerant	4.413	0.006	*Ascomycota*	*Pseudopeyronellaea*
	otu4	Tolerant	4.276	0.041	*Ascomycota*	*Pseudopeyronellaea*
	otu6	Tolerant	4.099	0.035	*Ascomycota*	*Pseudopeyronellaea*
	otu12	Tolerant	3.775	0.038	*Ascomycota*	*Pseudopeyronellaea*
	otu23	Susceptible	3.776	0.003	*Ascomycota*	Unclassified *Ascomycota*
	otu16	Susceptible	3.598	0.017	*Basidiomycota*	*Vishniacozyma*

### Microbial diversity and core microbiota of healthy and sour rot-affected grapes

#### Diversity and community composition from culture-independent analysis

**Fig 5:**
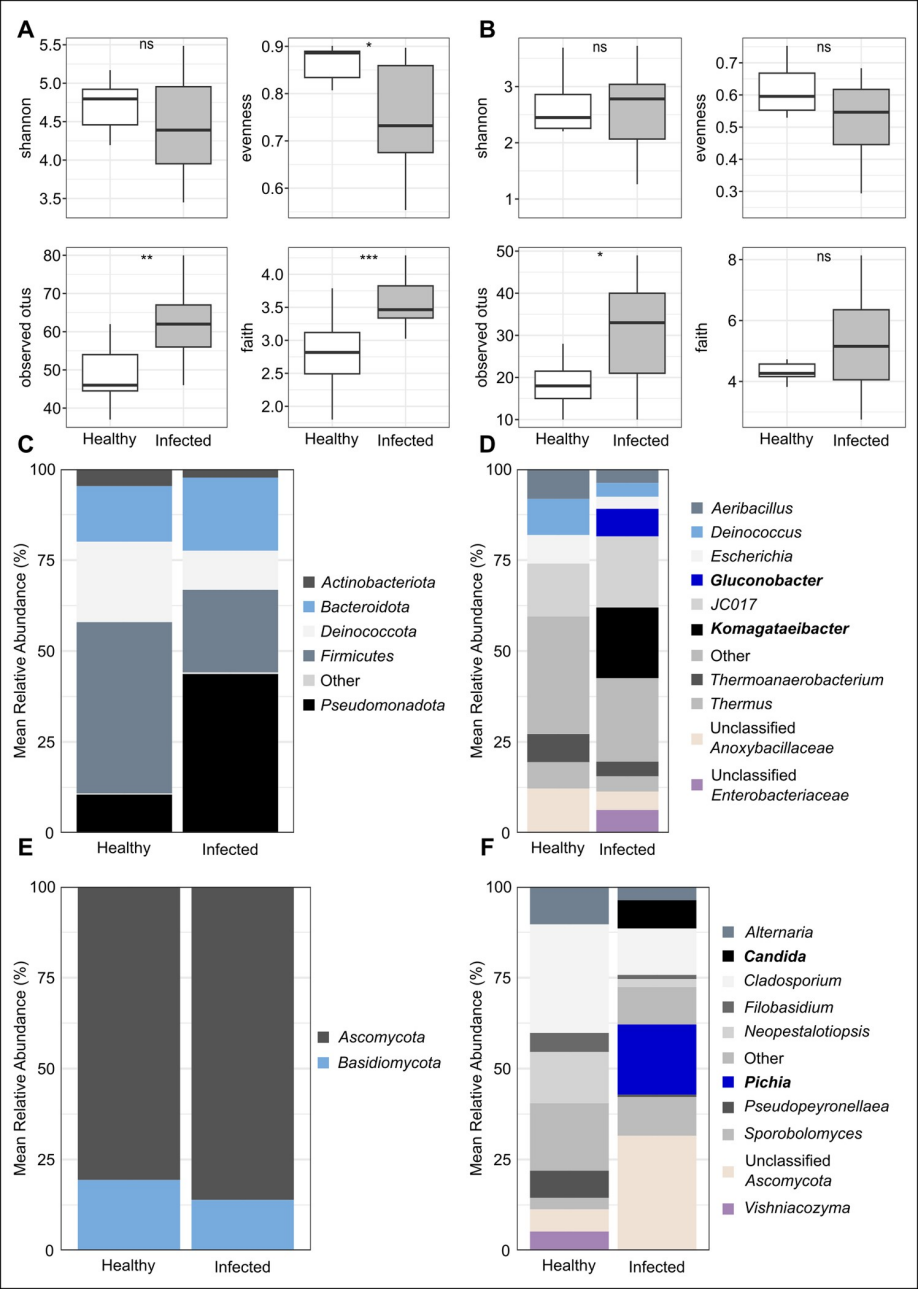
Microbial community diversity and OTU abundance differed between healthy and infected grape berries. *(A)* Bacterial diversity between healthy and infected berries. *(B)* Fungal diversity between healthy and infected berries. *(C)* Abundance of bacterial phyla between berry types. *(D)* Abundance of bacterial genera based on berry status *(E)* Abundance of fungal phyla between berry types; and *(F)* Abundance of fungal genera based on berry status.

As above, the microbial diversity of bacterial and fungal communities between healthy and sour rot-affected grape berries was calculated using Shannon, Evenness, Observed OTUs, and Phylogenetic diversity indexes as shown in Table 4. The average number of observed OTUs for bacterial communities per sample was 56 and 25 for fungal communities. The results indicated that the species diversity of bacteria did not differ significantly among samples within each health status (Table 4; Fig 5A, Kruskal Wallis test, *p = 0*.*168*). However, species were evenly distributed across samples and the number of observed species and phylogenetic diversity differed significantly between healthy and infected samples (Table 4; Fig 5A). We found no differences in species diversity, evenness, and phylogenetic diversity of fungi between healthy and sour rot-affected berries (Table 4; Fig 5B, Wilcoxon rank sum test, *p > 0*.*05*). Whereas, the number of observed species differed significantly (Kruskal Wallis test, *p = 0*.016*) between healthy and sour rot-affected samples.

**Table 4:** Analysis of variance among different healthy and infected berries of *Vitis vinifera*.

Gene	Diversity Index				Treatment
	Evenness	Shannon	Faith	Observed OTUs	
ITS	*KW = 1*.*900; p =* .*168*	*F*_*1*,*30*_ *=* .*203; p =* .*655*	*KW = 2*.*225; p =* .*136*	***KW = 5*.*763; p =* .*016***	Healthy/Infected
16S	***KW = 6*.*306; p =* .*012***	*F*_*1*,*30*_ *= 1*.*031; p =* .*318*	***F***_***1*,*30***_ ***= 15*.*700; p =* .*000***	***F***_***1*,*30***_ ***= 13*.*920; p =* .*001***	Healthy/Infected

H = Healthy berries, I = Infected berries, KW = Kruskal-Wallis test

Microbiota community composition at the phylum and genus levels was assessed using stacked bar graphs. All genera that had a relative abundance below the threshold predefined to select for the top ten taxa were grouped as “Other”. Overall, there were 57 bacterial genera (including 7 unclassified) from 39 families (including 2 unclassified) within 7 phyla. The top five most abundant phyla in both healthy and sour rot-affected berries were *Actinobacteriota*, *Bacteroidota*, *Deinococcota*, *Firmicutes*, and *Pseudomonadota* (Fig 5C). *Actinobacteriota*, *Deinococcota*, and *Firmicutes* were more abundant in healthy berries, but *Pseudomonadota* dominated sour rot-affected berries. We also observed a slight increase in the abundance of *Bacteroidota* in infected berries (Fig 5C). Bacterial genera also varied in abundance both within and between samples. The top 10 most abundant bacterial genera included *Aeribacillus*, *Deinococcus*, *Escherichia*, *Gluconobacter*, *Marinilabiliaceae bacterium* (*JC017*), *Komagataeibacter*, *Thermoanaerobacterium*, *Thermus*, “Unclassified *Anoxybacillaceae*” and “Unclassified *Enterobacteriaceae*”. Apart from *JC017*, the genera decreased in abundance from healthy to infected berries (Fig 5D). Strikingly, there was a shift in bacterial community abundance influenced by *Gluconobacter* and *Komagataeibacter*. These two genera were rare in healthy berries and recovered only in infected ones (Fig 5D). There was also a high abundance of “Unclassified *Enterobacteriaceae*”in sour rot-affected berries.

A total of 46 fungal genera (including 10 unclassified) were distributed in 37 families (including 6 unclassified) distributed between the *Ascomycota* and *Basidiomycota*. Both *Ascomycota* and *Basidiomycota* were almost equally distributed in healthy and sour rot-affected berries (Fig 5E). However, there were slightly more *Ascomycota* in infected berries compared to healthy ones. Among the top 10 most abundant fungal genera were *Alternaria*, *Candida*, *Cladosporium*, *Filobasidium*, *Neopestalotiopsis*, *Pichia*, *Pseudopeyronellaea*, *Sporobolomyces*, *Vishniacozyma*, and “Unclassified *Ascomycota”*. The abundance of most of the fungal genera dropped from healthy to infected berries with *Vishniacozyma* becoming absent in infected berries (Fig 5F). As noted earlier for *Gluconobacter* and *Komagataeibacter*, *Candida*, *Pichia*, and “Unclassified *Ascomycota*” also imposed a major shift in fungal community composition between healthy and infected berries. These three fungal taxa were mostly recovered from infected berries and were rare in healthy berries (Fig 5F).

#### Beta diversity and linear discriminant analysis effect size (LEfSe) of microbiota assemblages in healthy and sour rot-affected berries

**Fig 6:**
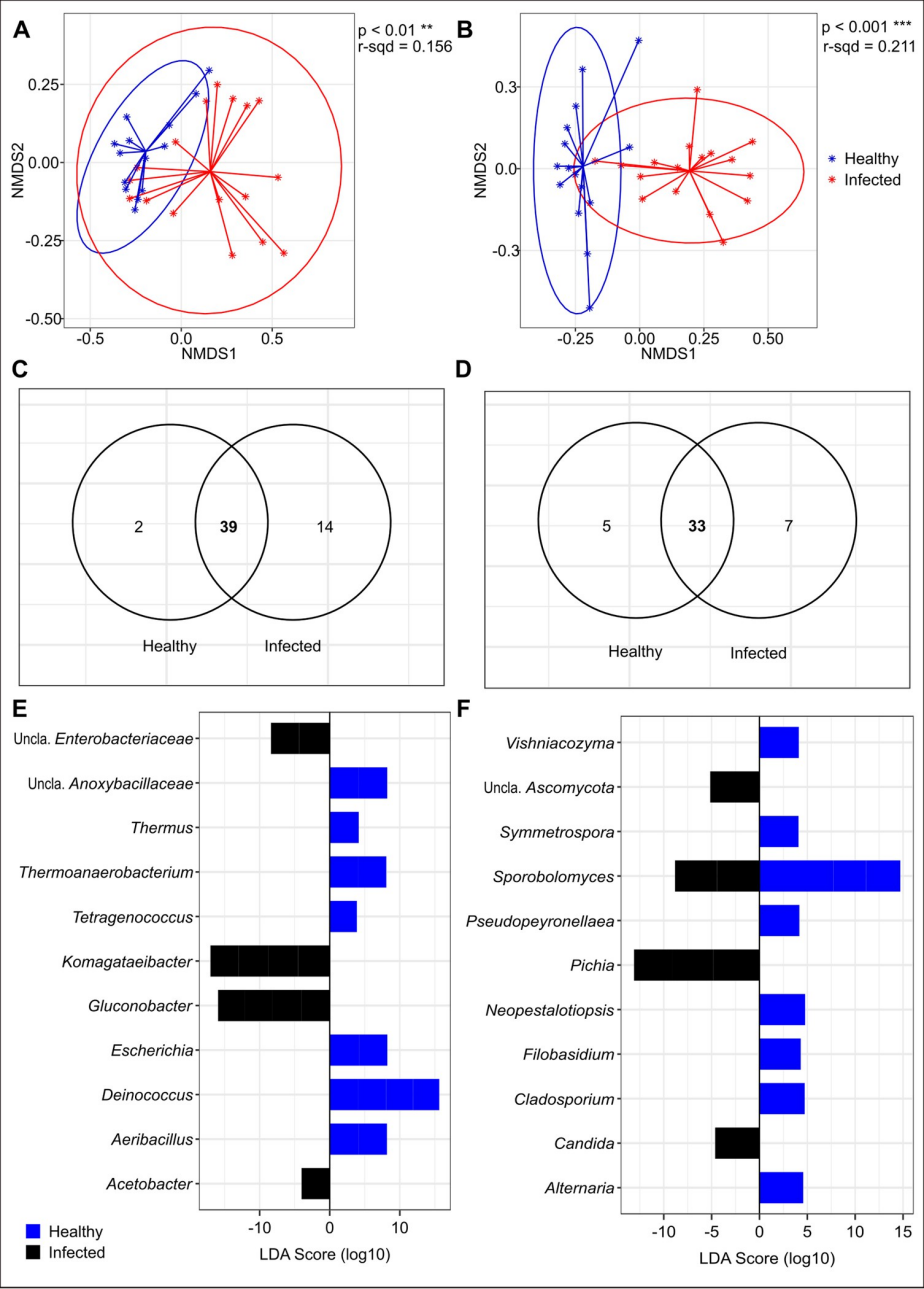
Microbiota community composition differed significantly between healthy and infected grape berries. *(A)* NMDS showing two clusters of bacteria communities between healthy and infected berries based on Bray-Curtis distances. *(B)* NMDS showing two distinct clusters of fungal communities between healthy and infected berries based on Bray-Curtis distances. *(C)* Distribution of bacterial OTUs between samples. *(D)* Distribution of fungal OTUs between samples. Linear discriminant analysis of significantly abundant *(E)* bacteria between healthy and infected berries; and *(F)* fungi between healthy and infected berries.

The bacterial and fungal community composition of culture-independent healthy and infected grape berries differed significantly. Following an analysis of dissimilarities, bacterial communities clustered according to berry health status using Bray-Curtis distances. The NMDS showed that the bacterial composition was different between healthy and infected berries (Fig 6A; Table 5). As observed for bacterial communities, fungal community composition was also significantly different between healthy and infected berries as samples clustered based on berry health status (Fig 6B; Table 5).

**Table 5:** Permutational multivariate analysis of variance for bacteria and fungi between healthy and sour rot-affected *Vitis vinifera* berries.

Gene	Factor	Degrees of Freedom	Sum of Squares	R^2^	F	*p*
16S	Berry Status	1	1.213	0.156	5.381	**0.001**
	Residual	29	6.538	0.843		
	Total	30	7.751	1.000		
ITS	Berry Status	1	2.063	0.211	8.002	**0.000**
	Residual	30	7.733	0.789		
	Total	31	9.796	1.000		

There were 55 core bacterial OTUs, with 39 of them overlapping between healthy and infected berries (Fig 6C). Two of the core bacterial OTUs belonging to *Neorhizobium* and *Stenotrophomonas* (S4 Table) were unique to healthy berries. Additionally, there were 14 unique OTUs in infected berries belonging to *Acetobacter*, *Actinomycetospora*, *Calditerricola*, *Corynebacterium*, *Gilliamella*, *Gluconobacter*, *Kineococcus*, *Komagataeibacter*, *Lacticaseibacillus*, *Patulibacter*, *Ramlibacter*, *Tatumella*, *Thermoanaerobacter*, and *Wolbachia* (S4 Table). On the other hand, 45 core fungal OTUs were recovered, 33 of which overlapped with healthy and infected berries (Fig 6D). Five OTUs belonging to *Aureobasidium*, *Leptobacillium*, *Mycocalicium*, *Periconia*, and *Wallemia* were unique to healthy berries. Similarly, seven OTUs were unique to infected berries including *Candida*, *Dissoconium*, *Ramularia*, *Saccharomycopsis*, *Starmerella*, “Unclassified *Fungi*”, and “Unclassified *Helotiales*” (S4 Table).

Following a LEfSe analysis, 25 bacterial OTUs were found to significantly differ in abundance between healthy and sour rot-affected berries. Of these, 14 OTUs belonging to the genera *Aeribacillus* (2 OTUs), *Deinococcus* (4 OTUs), *Escherichia* (2 OTUs), *Tetragenococcus*, *Thermoanaerobacterium* (2 OTUs), *Thermus*, and *“*Unclassified *Anoxybacillaceae”* (2 OTUs), were more abundant in healthy compared to infected berries (Fig 6E; Table 6). The remaining 11 OTUs associated with four genera including *Acetobacter*, *Gluconobacter* (4 OTUs), *Komagataeibacter* (4 OTUs), and “Unclassified *Enterobacteriaceae*” (2 OTUs), were significantly more abundant in infected than healthy berries (Fig 6E; Table 6).

**Table 6:** Linear discriminant analysis results showing significantly different taxa among healthy and infected grape berries.

OTU	Treatment	LDA	*p*-value	Phylum	Genus
Bacteria					
otu14	Healthy	4.109	0.003	*Firmicutes*	*Aeribacillus*
otu24	Healthy	4.058	0.002	*Firmicutes*	*Aeribacillus*
otu12	Healthy	4.036	0.008	*Deinococcota*	*Deinococcus*
otu19	Healthy	4.009	0.008	*Deinococcota*	*Deinococcus*
otu46	Healthy	3.838	0.017	*Deinococcota*	*Deinococcus*
otu76	Healthy	3.751	0.015	*Deinococcota*	*Deinococcus*
otu66	Healthy	4.182	0.003	*Proteobacteria*	*Escherichia*
otu69	Healthy	4.040	0.019	*Proteobacteria*	*Escherichia*
otu128	Healthy	3.874	0.030	*Firmicutes*	*Tetragenococcus*
otu5	Healthy	4.092	0.009	*Firmicutes*	*Thermoanaerobacterium*
otu20	Healthy	3.978	0.021	*Firmicutes*	*Thermoanaerobacterium*
otu3	Healthy	4.140	0.022	*Deinococcota*	*Thermus*
otu18	Healthy	4.157	0.019	*Firmicutes*	Unclassified *Anoxybacillaceae*
otu25	Healthy	4.045	0.011	*Firmicutes*	Unclassified *Anoxybacillaceae*
otu36	Infected	4.019	0.001	*Proteobacteria*	*Acetobacter*
otu93	Infected	4.003	0.001	*Proteobacteria*	*Gluconobacter*
otu115	Infected	4.233	0.000	*Proteobacteria*	*Gluconobacter*
otu142	Infected	3.911	0.001	*Proteobacteria*	*Gluconobacter*
otu195	Infected	3.787	0.001	*Proteobacteria*	*Gluconobacter*
otu13	Infected	4.495	0.000	*Proteobacteria*	*Komagataeibacter*
otu33	Infected	4.240	0.001	*Proteobacteria*	*Komagataeibacter*
otu73	Infected	4.291	0.000	*Proteobacteria*	*Komagataeibacter*
otu147	Infected	3.997	0.001	*Proteobacteria*	*Komagataeibacter*
otu77	Infected	4.361	0.000	*Proteobacteria*	Unclassified *Enterobacteriaceae*
otu95	Infected	4.027	0.001	*Proteobacteria*	Unclassified *Enterobacteriaceae*
**Fungi**					
otu9	Healthy	4.571	0.011	*Ascomycota*	*Alternaria*
otu3	Healthy	4.722	0.047	*Ascomycota*	*Cladosporium*
otu12	Healthy	4.311	0.018	*Basidiomycota*	*Filobasidium*
otu6	Healthy	4.769	0.049	*Ascomycota*	*Neopestalotiopsis*
otu29	Healthy	4.168	0.037	*Ascomycota*	*Pseudopeyronellaea*
otu37	Healthy	3.836	0.000	*Basidiomycota*	*Sporobolomyces*
otu41	Healthy	3.891	0.000	*Basidiomycota*	*Sporobolomyces*
otu122	Healthy	3.418	0.000	*Basidiomycota*	*Sporobolomyces*
otu124	Healthy	3.569	0.001	*Basidiomycota*	*Sporobolomyces*
otu30	Healthy	4.075	0.001	*Basidiomycota*	*Symmetrospora*
otu22	Healthy	4.110	0.029	*Basidiomycota*	*Vishniacozyma*
otu4	Infected	4.629	0.000	*Ascomycota*	*Candida*
otu2	Infected	4.812	0.000	*Ascomycota*	*Pichia*
otu5	Infected	4.303	0.000	*Ascomycota*	*Pichia*
otu10	Infected	3.993	0.000	*Ascomycota*	*Pichia*
otu7	Infected	4.423	0.000	*Basidiomycota*	*Sporobolomyces*
otu8	Infected	4.402	0.000	*Basidiomycota*	*Sporobolomyces*
otu1	Infected	5.142	0.000	*Ascomycota*	Unclassified *Ascomycota*

We found 18 fungal OTUs that differed significantly in abundance between healthy and sour rot-affected berries. Among these, 11 OTUs in the genera *Alternaria*, *Cladosporium*, *Filobasidium*, *Neopestalotiopsis*, *Pseudopeyronellaea*, *Sporobolomyces* (4 OTUs), *Symmetrospora*, and *Vishniacozyma* were more abundant in healthy berry samples (Fig 6F; Table 6). Whereas, the remaining 7 OTUs belonging to the genera *Candida*, *Pichia* (3 OTUs), *Sporobolomyces* (2 OTUs), and “Unclassified *Ascomycota*” were more abundant in sour rot-affected berries.

#### Diversity and community composition of microbiomes from culture-dependent analysis

**Fig 7:**
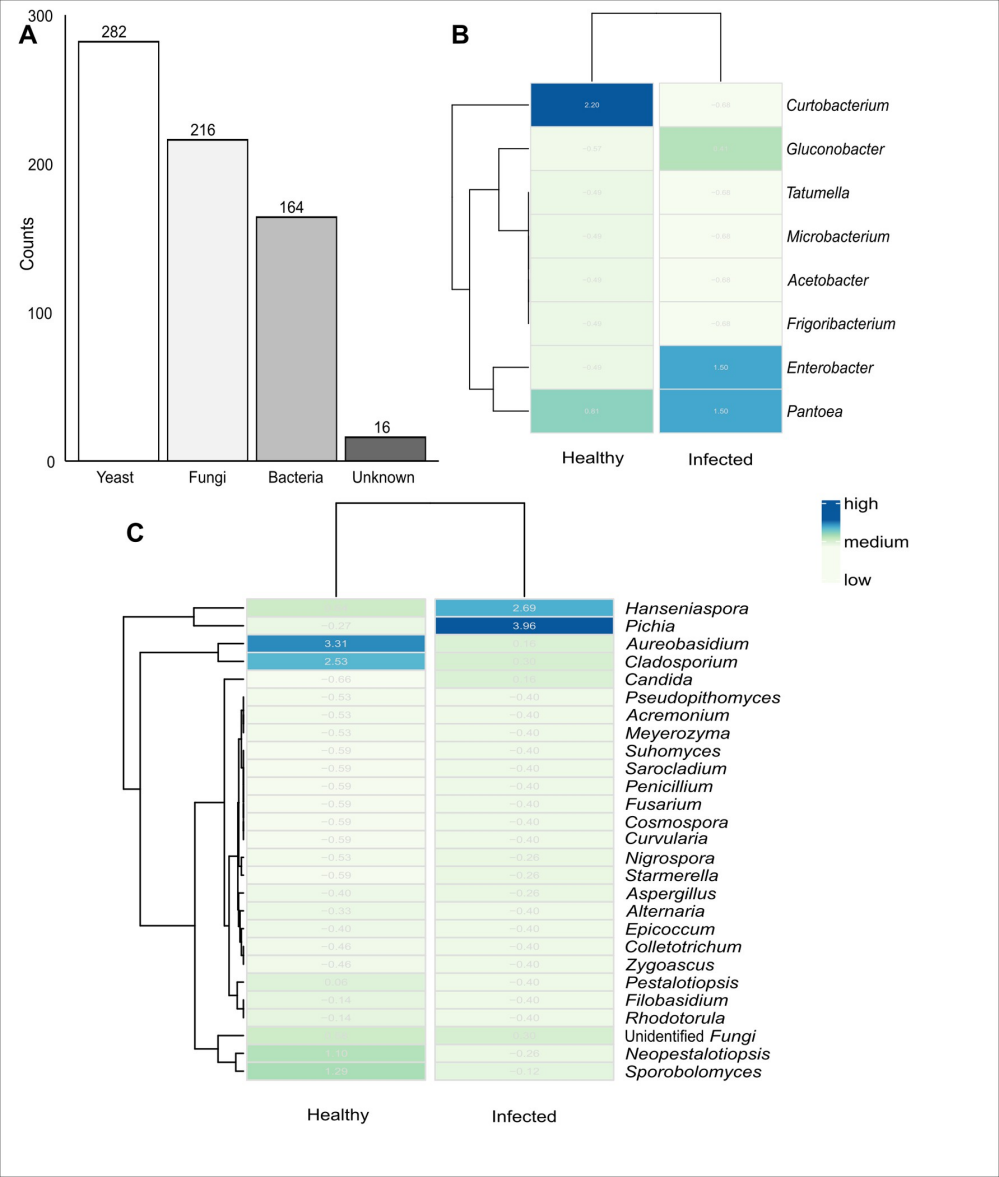
Culturable bacteria and fungi from healthy and infected grape berries. *(A)* Bacteria, fungi, and yeast obtained from culturing (counts represent the number of isolates cultured). *(B)* Abundance of cultured bacteria in healthy and infected berries; and *(C)* Abundance of cultured fungi in healthy and infected berries.

Among the ~700 bacterial and fungal isolates obtained from Sanger-based sequencing (Fig 7A), there were eight main bacterial genera from three phyla, including *Actinomycetota*, *Pseudomonadota*, and *Thermoproteota* (S2 Fig). The major genera from cultivation included *Acetobacter*, *Curtobacterium*, *Enterobacter*, *Frigoribacterium*, *Gluconobacter*, *Microbacterium*, *Pantoea*, and *Tatumella* (Fig 7B). Among these, *Acetobacter*, *Curtobacterium*, *Frigoribacterium*, *Microbacterium*, and *Tatumella* were only isolated from healthy berries, while *Gluconobacter* was only isolated from infected berries. On the other hand, *Pantoea* and *Enterobacter* were isolated from both healthy and infected berries (Fig 7B). More than 20 fungal genera belonging to the *Ascomycota* and *Basidiomycota* (S2 Fig) were successfully cultivated (Fig 7C). The top 10 main fungal genera cultivated in order of decreasing abundance included *Aureobasidium*, *Cladosporium*, *Hanseniaspora*, *Pichia*, *Sporobolomyces*, *Neopestalotiopsis*, “Unidentified *Fungi*”, *Pestalotiopsis*, *Filobasidium*, and *Rhodotorula* (Fig 7C). *Acremonium*, *Alternaria*, *Colletotrichum*, *Cosmospora*, *Curcularia*, *Epicoccum*, *Filobasidium*, *Fusarium*, *Meyerozyma*, *Penicillium*, *Pestalotiopsis*, *Pseudopithomyces*, *Rhodotorula*, *Sarocladium*, *Suhomyces*, and *Zygoascus* were only isolated from healthy berries. On the contrary, *Candida* was only isolated from infected berries. The rest of the genera were isolated from both healthy and infected berries including *Pichia* (Fig 7C).

#### Core microbiota associated with grape sour rot-affected grape berries

To identify the core microbial community associated with GSR, we compared the microbiota composition of healthy and sour rot-affected grapes. The eight bacterial genera isolated included 17 species from healthy and infected berries (Table 7). Overall, nineteen unidentified isolates (three *Curtobacterium* spp. and sixteen *Pantoea* sp.) were recovered from the berries. Among the bacterial microbiotas isolated from healthy berries was *Acetobacter persici*, a member of the acetic acid-producing bacterial group. One isolate of a non-AAB (*Pantoea vagans*) was also isolated from sour rot-affected berries. There were 38 fungal species from the 26 genera, constituting the fungal mycobiomes of healthy and infected berries (Table 7). Several unidentified species in the genera *Aspergillus*, *Aureobasidium*, *Candida*, *Cladosporium*, *Hanseniaspora*, *Neopestalotiopsis*, *Nigrospora*, *Pichia*, *Sporobolomyces*, *Starmerella*, *Suhomyces*, *Zygoascus* and “Unidentified *Fungi*” were also isolated from infected berries (Table 7).

**Table 7:** Bacterial and fungal species from cultural studies indicating berry status from which they were isolated. Empty cells (in gray color) indicate zero abundance for bacteria or fungi in healthy and infected berries.

Bacteria	Healthy	Infected
*Acetobacter persici*	1	
*Curtobacterium ammoniigenes*	1	
*Curtobacterium citreum*	8	
*Curtobacterium herbarum*	3	
*Curtobacterium luteum*	1	
*Curtobacterium oceanosedimentum*	13	
*Curtobacterium pusillum*	5	
*Curtobacterium* sp.	3	
*Enterobacter agglomerans*	1	2
*Frigoribacterium endophyticum*	1	
*Gluconobacter cerevisiae*		1
*Microbacterium testaceum*	1	
*Pantoea brenneri*	1	
*Pantoea eucrina*	1	
*Pantoea* sp.	15	1
*Pantoea vagans*		1
*Tatumella saanichensis*	1	
**Fungi**		
*Acremonium* sp.	2	
*Alternaria* sp.	5	
*Aspergillus* sp.	4	1
*Aureobasidium pullulans*	38	2
*Aureobasidium* sp.	23	2
*Candida californica*		1
*Candida* sp.		3
*Cladosporium devikae*	1	
*Cladosporium* sp.	48	5
*Colletotrichum fioriniae*	3	
*Cosmospora* sp.	1	
*Curvularia* sp.	1	
*Epicoccum italicum*	2	
*Epicoccum* sp.	2	
*Filobasidium floriforme*	7	
*Filobasidium magnum*	1	
*Fusarium* sp.	1	
*Hanseniaspora lachancei*		1
*Hanseniaspora* sp.	20	16
*Hanseniaspora uvarum*		5
*Meyerozyma caribbica*	2	
*Neopestalotiopsis dendrobii*	1	
*Neopestalotiopsis hispanica*	2	
*Neopestalotiopsis* sp.	24	1
*Nigrospora philosophiae-doctoris*	2	
*Nigrospora* sp.		1
*Penicillium* sp.	1	
*Pestalotiopsis pinicola*	3	
*Pestalotiopsis* sp.	7	
*Pestalotiopsis telopeae*	1	
*Pichia* sp.	6	31
*Pseudopithomyces* sp.	2	
*Rhodotorula mucilaginosa*	1	
*Rhodotorula* sp.	7	
*Sarocladium terricola*	1	
*Sporobolomyces* sp.	30	2
*Starmerella* sp.	1	1
*Suhomyces* sp.	1	
Unidentified Fungus	19	5
*Zygoascus* sp.	3	

**Fig 8:**
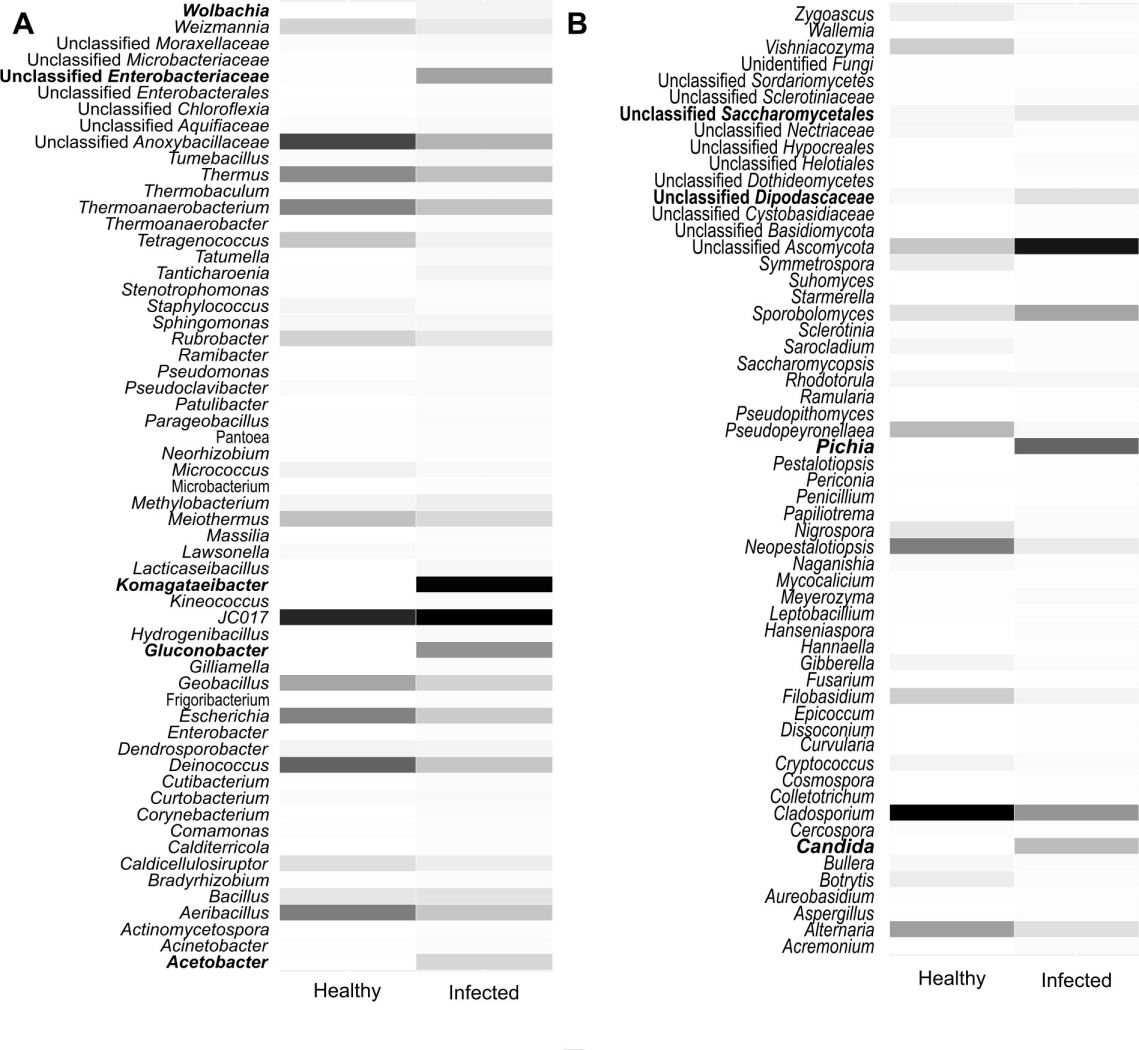
Core microbiomes associated with sour-affected grapes. *(A)* Core bacterial community members; and *(B)* Core fungal community members. Note: abundance/dominance is indicated by the color of the bands on the heatmap. Darker bands indicate higher dominance.

After pooling and analyzing the culture-dependent and independent data, the main bacteria genera that were consistently associated with infected berries included *Acetobacter*, *Gluconobacter*, *Komagataeibacter*, and “Unclassified *Enterobacteriaceae*“. *Tanticharoenia* and *Wolbachia* were also present in lower numbers (Fig 8A). For fungi, the major genera that appeared to be abundantly associated with sour rot-affected grapes included *Candida* and *Pichia* (Fig 8B). We also observed a high abundance of “Unclassified *Ascomycota*”, “Unclassified *Dipodascaceae*” and “Unclassified *Saccharomycetales*” in infected berries. The microbiotas in Fig 8 together with the isolated microbial species from healthy and infected berries (indicated in Table 4) constituted the overall core microbiomes of the sour rot complex.

## Discussion

Wine grapes are a vital economic crop that harbors microbes with critical roles for wine production [21]. The present study is the first to explore and compare changes in microbiota diversity and composition between GSR-susceptible (Vidal Blanc) and tolerant (Cabernet Franc) *Vitis vinifera* varieties in Mid-Atlantic vineyards, using culture-dependent and independent techniques. We investigated the effect of insecticide application and netting treatment on the microbiomes of the two varieties and identified the core microbial assemblages of healthy and sour rot-affected grape berries. The number of unique sequences for the 16S and ITS datasets used for this study ranged from 843 to 1,406 sequences. Approximately 700 fungi and bacteria combined were isolated belonging to diverse taxa. We found that microbiome alpha diversity did not differ between healthy berries of tolerant and susceptible grape varieties. However, there were differences in the fungal community composition between healthy berries of tolerant and susceptible grape varieties. Bacterial alpha diversity differed between healthy and infected berries (except for Shannon index) while fungal diversity did not differ between berry types for most of the alpha diversity indexes except for the number of observed OTUs. The community composition of both bacteria and fungi differed significantly between healthy and sour rot-affected grape berries.

Our results indicated that bacterial and fungal diversity did not differ between healthy Mustang Max-treated and untreated berries. This was true for net-protected and unprotected berries. These results corroborate those of Barata et al. [35]. In a study to evaluate the role of insects in sour rot development, Barata et al. revealed that all protected and unprotected bunches had similar species diversities [35]. Among some of the abundant fungal species from their study were *Aureobasidium pullulans*, *Candida zemplinina Cryptococcus flavescens*, *Cryptococcus laurentii*, *Rhodotorula spp*., and *Sporobolomyces roseus*. Similarly, some of the abundant species we found from net-protected and unprotected berries included *Aureobasidium pullulans* and many undescribed *Candida*, *Gibberella*, *Filobasidium*, *Hanseniaspora*, *Pichia*, *Pseudopeyronellaea*, *Rhodotorula*, and *Sporobolomyces* species. There was no variation in microbiota alpha diversity among varieties. While there is adequate data on the diversity of the microbiota of grapes and wine, there is limited information regarding the effect of insecticides on grape microbiota. A similar study found that when fields were treated with chlorantraniliprole, differences in soil bacterial diversity were detected between the heading and ripening stages of rice, but differences in diversity within each growing stage were not observed [28]. These results demonstrated that resident bacterial and fungal communities are resilient to the tested treatments. When Knorr et al. tested different fungicide doses at different time intervals, they found that fungal content between treatments remained relatively constant but samples from treated and untreated plots were separated in a principal component analysis [26]. The findings from this study align with the latter two previous research studies.

In the current study, we did not observe differences in the community composition of bacterial phyla and genera (OTUs) between netting, insecticide as well as varietal treatments. Although *Firmicutes*, *Deinococcota*, *Pseudomonadota* (syn. *Proteobacteria*), *Actinobacteriota*, and *Bacteroidota* dominated the bacterial microbiomes in both tolerant and susceptible varieties, these differences were not significant as demonstrated by linear discriminant analysis (LDA) results. Zhang et al. reported differences between bacterial taxa in their study [47]. They reported the abundance of *Firmicutes* and *Pseudomonadota* in grape must of clones from the same variety [47]. The differences in fungal communities were not significant for insecticide and netting treatments. However, the composition of fungal taxa differed between varieties in our study. This may be an indication that the disease-resistant variety could be harboring specific microbiomes that confer resistance. Further investigation is needed to ascertain this observation. In an investigation to elucidate the effect of penconazole on grape phyllopshere, Perazzolli et al. reported no differences in the proportion of bacterial and fungal phyla between treatments [25]. Rather, they found differences in these phyla by grapevine location.

In the current study, the bacterial genus *Meiothermus*, was more abundant in Mustang Max-treated and unprotected berries for both varieties. Perazzolli et al. saw a similar trend between fungicide treated and untreated grapevine samples [25]. When a biofungicide (AZ78) was sprayed on grapevines, the abundance of *Deinococcus* reduced [25]. While these authors reported a high abundance of *Alternaria* in penconazole-untreated grapes, the scenario was not obvious in our study. *Cladosporium* was more abundant in -Max and -Net berries for both varieties. However, when netting was used, the abundance of *Pseudopeyrollaea*, “Unclassified *Ascomycota*”, Unclassified *Saccharomycetales*”, and *Vishniacozyma* increased especially in the susceptible variety. *Cladosporium* was the most abundant fungus across varieties and treatments. Bokulich et al. found *Cladosporium* to be most abundant in a 2010 grape must from a vineyard in California [56], while Porter et al. consistently isolated *Lachancea fermentati* from the susceptible grape variety Vidal Blanc [57]. When treated with tebuconazole, the abundance of potentially beneficial phyllospheric *Dothideomycetes* and *Sordariomycetes* on powdery mildew-infected cucumber leaves reduced [34].

In a comparative analysis between healthy and sour rot-affected grape berries, we found that species diversity of bacteria differed significantly among samples. There was no difference in fungal alpha diversity among samples except for the number of species. The observed results for bacterial communities are consistent with previous studies. For instance, Gao et al. reported differences in bacterial communities between healthy and infected pepper plants [17]. Hall et al. found *Acetobacteraceae* to be common in both healthy and sour rot-affected berries [19]. We observed the recurrence of *Deinococcota*, *Firmicutes*, and *Pseudomonadota* (containing *Acetobacteraceae*) in both healthy and infected berries although in varying abundances. Hall et al. concluded in their study that grape berries had similar bacterial and fungal microbiota, irrespective of the presence of sour rot symptoms [19]. We found differences in the bacterial and fungal communities between healthy and sour rot-affected berries. For instance, we found no *Candida* in healthy berries while several yeasts including *Filobasidium*, *Meyerozyma*, *Nigrospora*, *Pseudopithomyces*, *Rhodotorula*, *Suhomyces*, and *Zygoascus* were only found in healthy berries. Although *Actinobacteriota*, *Deinococcota*, and *Firmicutes* were most abundant in healthy berries, there was an observed shift to *Pseudomonadota* in sour rot-affected berries.

*Pseudomonadota* hosts most of the currently known sour rot causing bacteria like *Acetobacter* and *Gluconobacter*. Many of the bacterial reads from our culture data belonged to *Curtobacterium*, as found by other authors [25]. Hall et al. in their research found *Pseudomonas* to be the most abundant bacterial genus from culture data [19]. As seen for phylum *Pseudomonadota*, the genera *Gluconobacter* and *Komagataeibacter* dominated infected berries relative to the healthy counterparts. Additionally, bacterial genera decreased in abundance from healthy to infected berries, except for *JC017* and “Unclassified *Enteobacteriaceae*”. Hall et al. also observed a shift in bacterial communities as acetic acid bacteria (AAB) became more prevalent in sour rot-affected berries [19]. Furthermore, Hall et al. found that *Acetobacter* dominated sour rot-affected grapes. In the culture-independent techniques of the current study, sour rot-affected grapes were dominated by *Komagataeibacter*. This study is the first to report *Komagataeibacter* from sour rot infected grape berries. This bacterium is reported to produce acetic acid and has been found in abundance from a fermented grape must [58, 59]. This indicates that *Komagataeibacter* may play a critical role in the observed symptoms of sour rot in regional vineyards. Strains of this bacterium thrive in acetic acid-rich environments and are nutritionally demanding and difficult to cultivate in artificial media [60]. This could explain our inability to isolate this bacterium in culture. The involvement of *Gluconobacter* and *Acetobacter* in sour rot disease development has been demonstrated in other studies [23, 35].

Regarding fungi, there was no observable difference in abundance of fungal phyla between healthy and infected berries. As noted for bacteria, there was a decrease in abundance of fungal genera from healthy to infected berries. *Alternaria*, *Cladosporium*, *Filobasidium*, *Neopestalotiopsis*, *Pseudopeyronellaea*, and *Sporobolomyces* decreased in abundance while *Candida* and *Pichia* became more abundant in infected berries. There was also a considerable amount of “Unclassified *Ascomycota*” in infected berries. In a study to understand the ecological interaction between grape berry microorganisms and *Drosophila* flies during GSR development, Barata et al. found that *Candida* and *Hanseniaspora* species dominated sour rot-affected berries [35]. They also reported that *Aureobasidium pullulans* was more abundant in healthy berries compared to their sour rot-affected counterparts. These findings were corroborated by the current study. In addition to *A*. *pullulans*, *Filobasidium floriforme*, *Rhodotorula* sp., *Sporobolomyces* sp., and *Zygoascus* sp. were among the cultured yeasts that dominated healthy berries in our study. These yeasts have not been previously reported to be involved in sour rot development of wine grapes. Our results were also consistent with Hall et al. who reported a high abundance of *Pichia* spp. in sour rot-affected versus healthy berries [19].

We observed that most of the cultured yeasts were recurrent in both healthy and infected berries, but this was not the case for most of the cultured bacteria. Apart from *Pantoea* and *Enterobacter*, the rest of the culturable bacterial taxa were either found only in healthy or infected berries. When Shi et al. investigated the effect of crop type and fungicide treatment on phyllosphere microbiome, they found that bacterial composition was different between crop species [61]. In another study, there were observed differences in bacterial and fungal communities between healthy and infected pepper plants [17].

We successfully identified core bacteria and fungi from the combined analyses of culture-dependent and independent data. *Acetobacter*, *Gluconobacter*, and *Komagataeibacter* were the dominant genera among core microbiomes from infected berries relative to healthy ones, as found in similar studies [19, 35, 58]. Although *Acetobacter* is an AAB, we isolated a culture from healthy berries. Since *Acetobacter* is mostly known to be associated with infected berries, it might have been introduced on the healthy berry surface by feeding fruit flies. *Tanticharoenia* and *Wolbachia* were also seen in lower abundance. These two bacterial genera are reported associates of insect guts in other studies [62, 63] and are thus adapted to both plant and insect hosts. *Tanticharoenia* and *Wolbachia* have not been previously reported to be associated with sour rot development and would therefore be good candidates for *in vitro* assays. Certain filamentous fungi that were recovered from both healthy and infected berries have been reported to be involved with sour rot development elsewhere. For example, *Aspergillus carbonarius*, was seen to cause sour rot of table grapes (*Vitis vinifera*) in California [15]. *Botrytis cinerea* and *Erisiphe necator* also cause damage to grape berry skin [64–66], thus exposing berry pulp to causal agents and facilitating sour rot development.

In conclusion, netting, insecticide, and variety had no discernible impact on the microbiota diversity of healthy grape berries. Given the demonstrated role of microbiomes in the development of wine flavors [67] and other enological characteristics, the observed minimal impact of insecticide treatment on resident microbiota would benefit growers during disease management. There was a difference in fungal composition between varieties. This change was driven mainly by yeasts, a group of microbiomes that are known for their role in fermentation. We found differences in microbiota community composition between healthy and sour rot-affected grape berries. Furthermore, there was a major shift in bacterial and fungal communities as AAB and yeast dominated infected berries. The observed increase in abundance of yeasts and AAB could be indicative of their ability to effectively displace other bacterial and fungal taxa on the infected berry phylloplane as grapes ripen. The production of alcohol and acetic acid by yeast and bacteria during sour rot development could alter the berry pH and therefore reduce the population of less tolerant microbiota in favor of the tolerant counterparts. Certain bacterial and fungal core members were seen to overlap between healthy and infected berries. These shared core microbiotas are likely to be generalists and would likely play a negligible role in sour rot development. Furthermore, the observed unique OTUs (S4 Table) constitute the specialist group of microbiotas. We believe that unique OTUs from infected berries are key players in sour rot development that should be investigated further.

*Komagataeibacter* was found in high abundance in infected berries in relation to healthy berries and therefore adds to the ranks of *Acetobacter* and *Gluconobacter* as a potential driver in GSR development. Further studies will help to elucidate the potential for *Komagataeibacter* to cause sour rot symptoms. Other bacteria of the acetic acid-producing group isolated in abundance from infected berries included *Enterobacter agglomerans*, *Gluconobacter cerevisiae*, and *Pantoea vagans*. *E*. *agglomerans*, and *P*. *vagans* would also be good candidates for sour rot *in vitro* assays since they were consistently recovered in culture and from culture-independent techniques. This is true for yeasts like *Candida* spp. and *Pichia* spp. Although species of *Candida* and *Pichia* have previously been demonstrated to be involved in sour rot development, this study recovered several undocumented members of the two genera that would be worth exploring in the future. This study was not meant to capture the entire microbiome situation of Mid-Atlantic vineyards in relation to sour rot, but rather to lay a foundation for further investigations of this complex disease in the region.

## Supporting information

S1 Table Test of normality results for OTUs of samples from two grape varieties within one vineyard. The first set of gene pairs were tested for healthy berries only while the second pair included tests for samples from healthy and infected berries.
(XLSX)

S2 Table Sequence reads recovered for each sample. Experiment 1 represents sequence reads for insecticide and netting treatments on tolerant and susceptible varieties, while experiment 2 corresponds to reads from healthy and infected berries (infected berries are denoted by gsrXX). Experiment 2 included samples from three varieties, mainly Cabernet Franc, Vidal Blanc, and Merlot.
(XLSX)

S3 Table Permutational multivariate analysis of variance for bacteria and fungi between tolerant and susceptible *Vitis vinifera* varieties. (XLSX)

S4 Table Unique bacterial and fungal microbiotas in healthy and infected *Vitis vinifera* berries. Gray color indicates the presence of OTUs in different berry types.
(XLSX)

S1 Fig Alpha diversity plots for (A) Bacteria between tolerant and susceptible varieties; and (B) Fungi between tolerant and susceptible varieties. (Tole. Refers to tolerant variety and Susc. Refers to the susceptible variety).
(TIF)

S2 Fig Culture-based microbial phyla. *(A)* Bacteria and *(B)* Fungi. Pie chart shows that *Pseudomonadota* were the most abundant group cultured followed by *Actinomycetota*. *Ascomycota* dominated culturable fungi followed by *Basidiomycota*.
(TIF)

S1 Appendix Step-by-step summary from sample preparation to sequencing and analysis. (PDF)
