# Supplementary figures and images for: Comparative analysis of grape berry microbiota uncovers sour rot associates from a Maryland vineyard
Source: PLoS One. 2025 Feb 6;20(2):e0314397. doi: 10.1371/journal.pone.0314397 (PMC11801560; doi:10.1371/journal.pone.0314397)

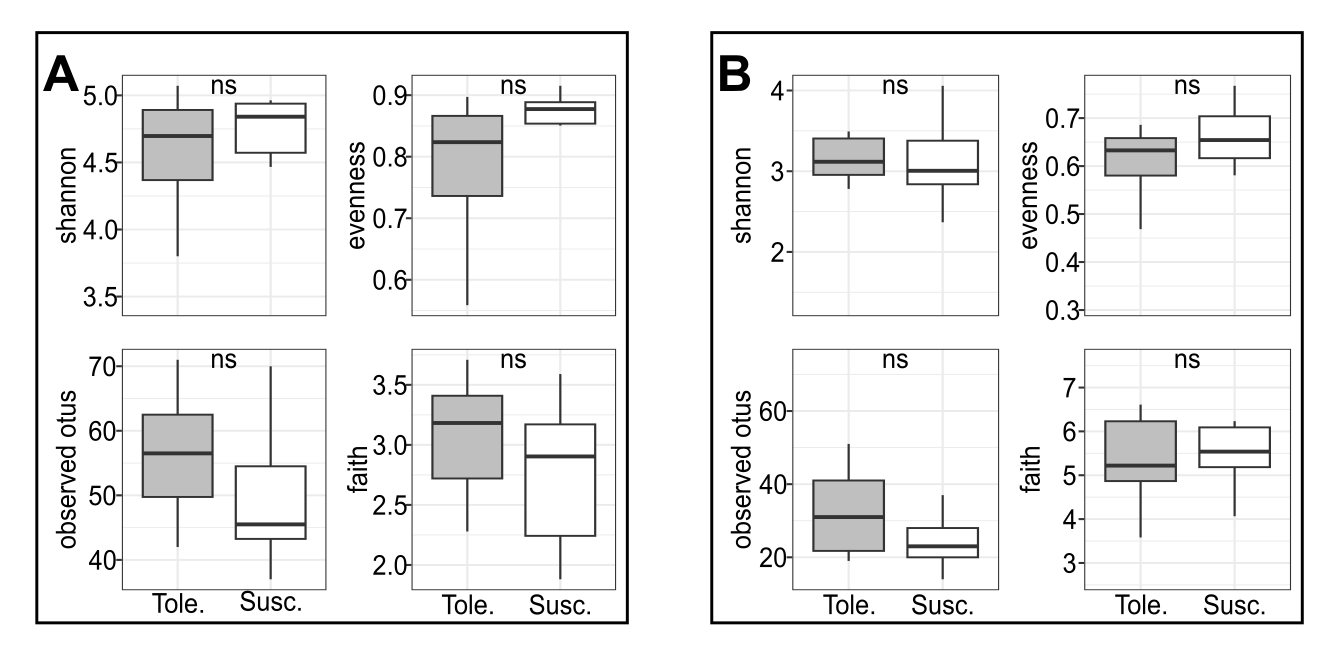

Supplement: S1 Fig — Alpha diversity plots for (A) Bacteria between tolerant and susceptible varieties; and (B) Fungi between tolerant and susceptible varieties. (Tole. Refers to tolerant variety and Susc. Refers to the susceptible variety). (TIF) [file pone.0314397.s005.tif]

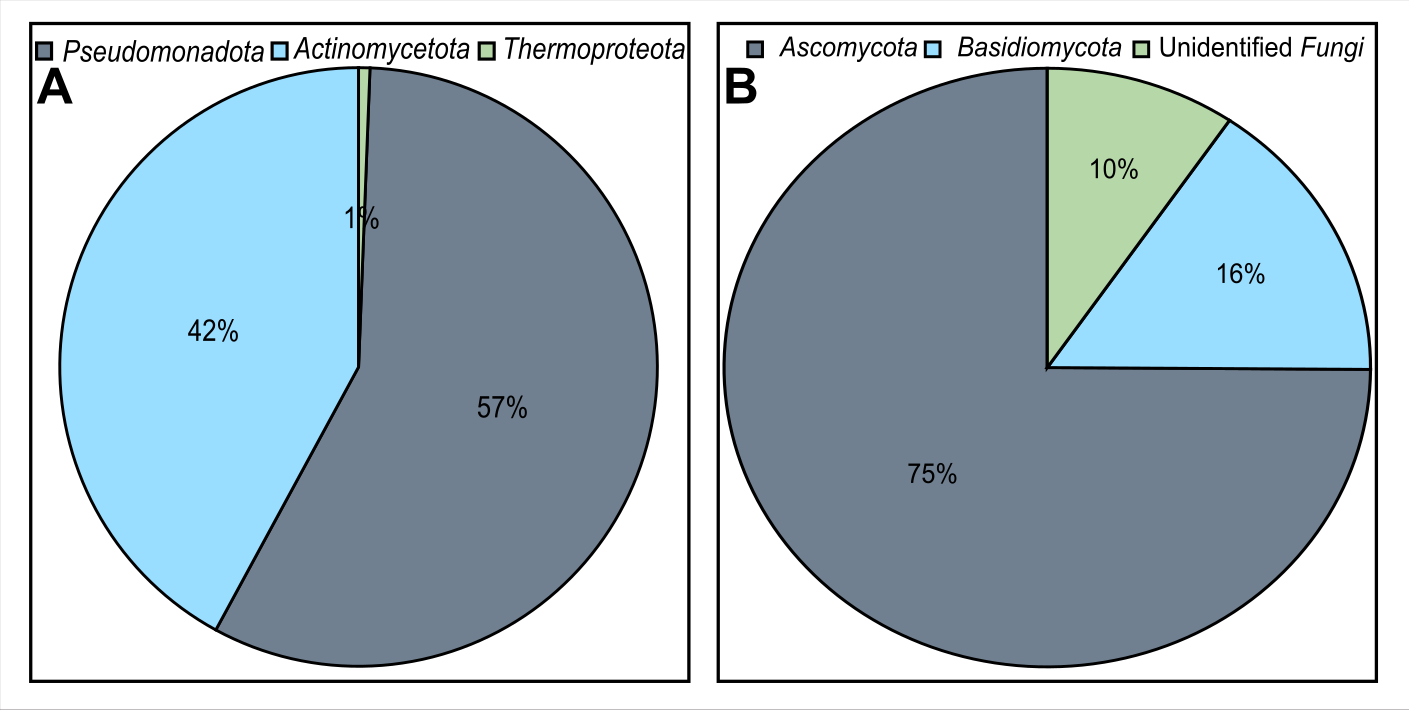

Supplement: S2 Fig — (A) Bacteria and (B) Fungi. Pie chart shows that Pseudomonadota were the most abundant group cultured followed by Actinomycetota. Ascomycota dominated culturable fungi followed by Basidiomycota. (TIF) [file pone.0314397.s006.tif]
