# Supplementary material for: Comparative analysis of grape berry microbiota uncovers sour rot associates from a Maryland vineyard
Source: PLoS One. 2025 Feb 6;20(2):e0314397. doi: 10.1371/journal.pone.0314397 (PMC11801560; doi:10.1371/journal.pone.0314397)
Supplement: S1 Appendix — (PDF) [file pone.0314397.s007.pdf]

Step-by-step summary from sample preparation to sequencing and analysis

Graphical summary

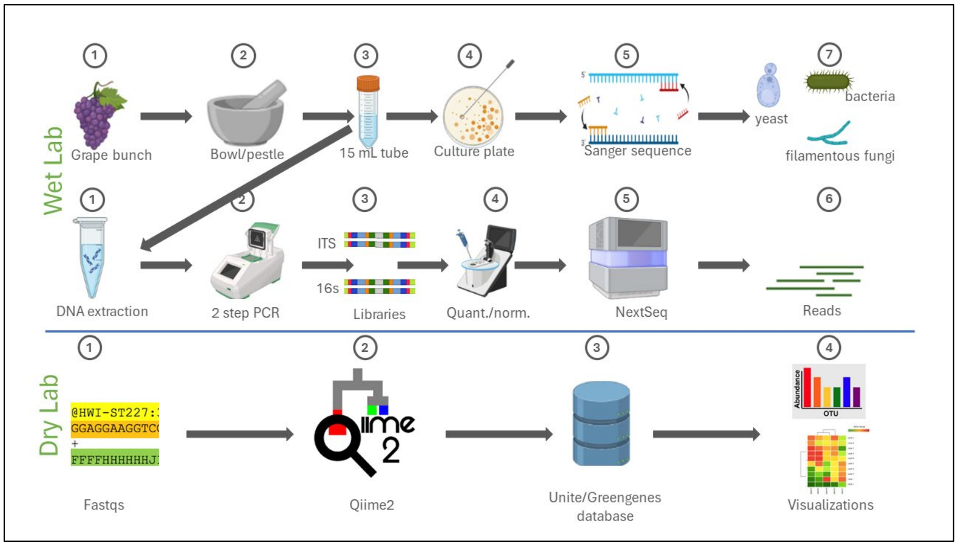

Summary of our research strategy from sampling, through processing, sequencing, and analysis (*image generated in https://www.biorender.com/*).

Preliminaries

The protocol includes primer sequences specific to bacterial V4 and fungal ITS1 regions respectively.

It also includes overhang adapter sequences that must be appended to the primer pair sequences for compatibility with Illumina index and sequencing adapters.

NB: Illumina does not sell these primers. We modified our 16s and ITS primers using these overhang adapters and ordered the modified primers from Genwiz.

Gene	Overhang	Locus-specific primer
ITS1f	TCGTCGGCAGCGTCAGATGTGTATAAGAGACAG	5`-CTTGGTCATTTAGAGGAAGTAA-3`
ITS2r	GTCTCGTGGGCTCGGAGATGTGTATAAGAGACAG	5`-GCTGCGTTCTTCATCGATGC-3`
515F	TCGTCGGCAGCGTCAGATGTGTATAAGAGACAG	5`-GTGCCAGCMGCCGCGGTAA-3`
806R	GTCTCGTGGGCTCGGAGATGTGTATAAGAGACAG	5`- GGACTACHVGGGTWTCTAAT-3`

For example, the ITS1f primer ordered was:

5`-TCGTCGGCAGCGTCAGATGTGTATAAGAGACAGCTTGGTCATTTAGAGGAAGTAA-3`

We ordered the **Nextera XT Index Kit v2 (96 indices-192 samples)** for the study and used the full complement of indices to pool together up to 96 libraries for sequencing.

MiSeq run output is approximately **> 20 million reads** and, assuming 96 indexed samples, can generate **> 100,000 reads per sample**, recognized as sufficient for metagenomic studies. For ITS1, a **2x150 cycle** generates **~15,000-100,1000 reads/sample**. However, we used the NextSeq 2000 machine for sequencing that generates way more reads per sample than the MiSeq instrument.

Taxonomic classification of 16s and ITS was done using **Greengenes and UNITE database classifiers** respectively. Other databases for 16s include **RDP and Silver**.

DNA extraction

DNA was extracted using Omega E.Z.N.A. HP Fungal DNA Kit (Omega Bio-tek, Inc., Georgia, USA) following the manufacturer's instructions with slight modifications. The DNA was concentrated in two ways (i) by eluting the DNA column with the same solution twice OR (ii) pooling together the two elutions from the protocol, drying these at 65 °C for 3-4 hours and then eluting with 30 uL of elution buffer as opposed to 100 uL recommended by the manufacturers.

Library prep workflow

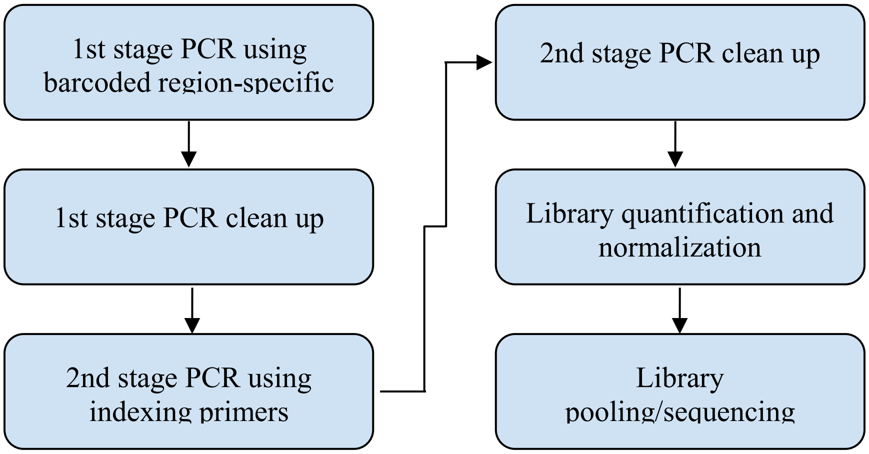

1. 1st Stage PCR

a) A 25 ul reaction was set as follows for each sample well:

Reagent	Volume
Mater mix	12.5 ul
Forward primer (10 uM)	1.0 ul
Reverse primer (10 uM)	1.0 ul
DNA template	1.0 ul
Millipore water	9.5 ul

b) Thermocycler conditions were:

**16s**	**ITS**
95 °C for 5 mins	94 °C for 60 secs
30 cycles of:	35 cycles of:
95 °C for 40 secs	94 °C for 30 secs
53 °C for 2 mins	53 °C for 30 secs
72 °C for 60 secs	68 °C for 30 secs
72 °C for 7 mins	68 °C for 10 mins
4 °C hold	4 °C hold

c) Gel electrophoresis to verify amplification. For some reason, there were a lot of primer dimers, but these did not affect the amplification when DNA concentration was above 10 ng/ul.

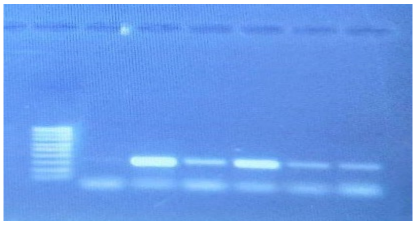

2. 1st Stage PCR clean-up

We used ExoSapIT to clean-up PCR products from free primers and primer dimers.

d) For this, 1 ul of molecular grade water was added onto 4 uL of PCR product were and 2 uL of ExoSap and the mixture was centrifuged in a Mini Centrifuge (Fisher Scientifc, Korea) for 10 sec and ran the following cycling conditions:

**Temperature**	**Time**
37 °C (1 cycle)	15 mins
80 °C (1 cycle)	15 mins
12 °C	Hold

3. 2nd Stage PCR

This is the ligation stage where dual indexes and Illumina sequencing adapters are attach together. We used the Nextera Index Kit v2 Set A and B for this purpose. Here, the products from the first PCR amplification are bound to the dual index adapters.

e) A 25 ul reaction for each sample well was set up as follows:

**Reagent**	**Volume**
Mater mix	12.5 ul
Forward primer (N7XX)	2.5 ul
Reverse primer (N5XX)	2.5 ul
DNA template	2.5 ul
Millipore water	5 ul

f) The forward and reverse indexes were paired in the following combinations as recommended by Illumina. The Illumina Experiment Manager (IEM) was used to generate the possible primer pairs that were used for sequencing.

N701/N502	N707/N502	N701/N503	N707/N503
N702/N502	N710/N502	N702/N503	N710/N503
N703/N502	N711/N502	N703/N503	N711/N503
N704/N502	N712/N502	N704/N503	N712/N503
N705/N502	N714/N502	N705/N503	N714/N503
N706/N502	N715/N502	N706/N503	N715/N503

g) The cycling conditions were as follows:

Conditions

95 °C for 3 mins

8 cycles of:

- 95 °C for 30 secs
- 55 °C for 30 secs
- 72 °C for 30 secs

72 °C for 4 mins

4 °C hold

h) Gel electrophoresis to verify ligation/indexing. This was done by loading the 1st and 2nd PCR products of each sample side-by-side to verify the size of the first and second bands. When ligation happens, the bands of the second PCR product should be higher than those of the first PCR.

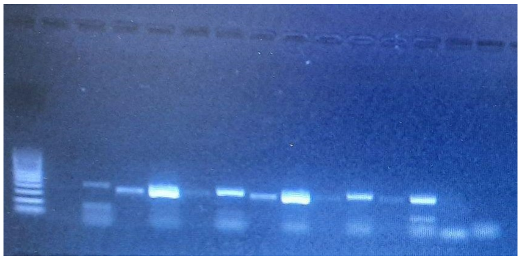

4. 2nd Stage PCR clean-up

i) Same as for the 1st stage PCR clean-up above.

5. Library quantification and normalization

j) Done using a NanoDrop machine and molecular grade water. The samples are diluted at almost the same concentration, say within the range of 400-500 ng/uL depending on the sample with the lowest concentration.

6. Library pooling for sequencing

k) Samples were pooled in proportionate amounts relative to the sample with the lowest concentration. For instance, for a sample with 500 ng/ul, 1 ul was pooled while for a sample with 400 ng/ul, ~ 1.25 ul was pooled. The pooled library was then normalized and sequenced on the NextSeq 2000.
